# Multimodal perturbation analyses of cyclin-dependent kinases reveal a network of synthetic lethalities associated with cell-cycle regulation and transcriptional regulation

**DOI:** 10.1038/s41598-023-33329-2

**Published:** 2023-05-11

**Authors:** Kyle Ford, Brenton P. Munson, Samson H. Fong, Rebecca Panwala, Wai Keung Chu, Joseph Rainaldi, Nongluk Plongthongkum, Vinayagam Arunachalam, Jarek Kostrowicki, Dario Meluzzi, Jason F. Kreisberg, Kristen Jensen-Pergakes, Todd VanArsdale, Thomas Paul, Pablo Tamayo, Kun Zhang, Jadwiga Bienkowska, Prashant Mali, Trey Ideker

**Affiliations:** 1 Department of Bioengineering, University of California San Diego, La Jolla, CA 92093 USA; 2 Department of Medicine, University of California San Diego, La Jolla, CA 92093 USA; 3 Biomedical Sciences Program, University of California San Diego, La Jolla, CA 92093 USA; 4 Pfizer Inc, 10555 Science Center Drive, San Diego, CA 92121 USA

**Keywords:** Cancer, Breast cancer, Cancer genetics, Cancer genomics, Oncogenes, Genetic interaction, Genomic engineering, Functional genomics, Gene expression, Gene regulation, Cell growth, Cell signalling, Sequencing

## Abstract

Cell-cycle control is accomplished by cyclin-dependent kinases (CDKs), motivating extensive research into CDK targeting small-molecule drugs as cancer therapeutics. Here we use combinatorial CRISPR/Cas9 perturbations to uncover an extensive network of functional interdependencies among CDKs and related factors, identifying 43 synthetic-lethal and 12 synergistic interactions. We dissect CDK perturbations using single-cell RNAseq, for which we develop a novel computational framework to precisely quantify cell-cycle effects and diverse cell states orchestrated by specific CDKs. While pairwise disruption of CDK4/6 is synthetic-lethal, only CDK6 is required for normal cell-cycle progression and transcriptional activation. Multiple CDKs (CDK1/7/9/12) are synthetic-lethal in combination with PRMT5, independent of cell-cycle control. In-depth analysis of mRNA expression and splicing patterns provides multiple lines of evidence that the CDK-PRMT5 dependency is due to aberrant transcriptional regulation resulting in premature termination. These inter-dependencies translate to drug–drug synergies, with therapeutic implications in cancer and other diseases.

## Introduction

**Figure 1:**
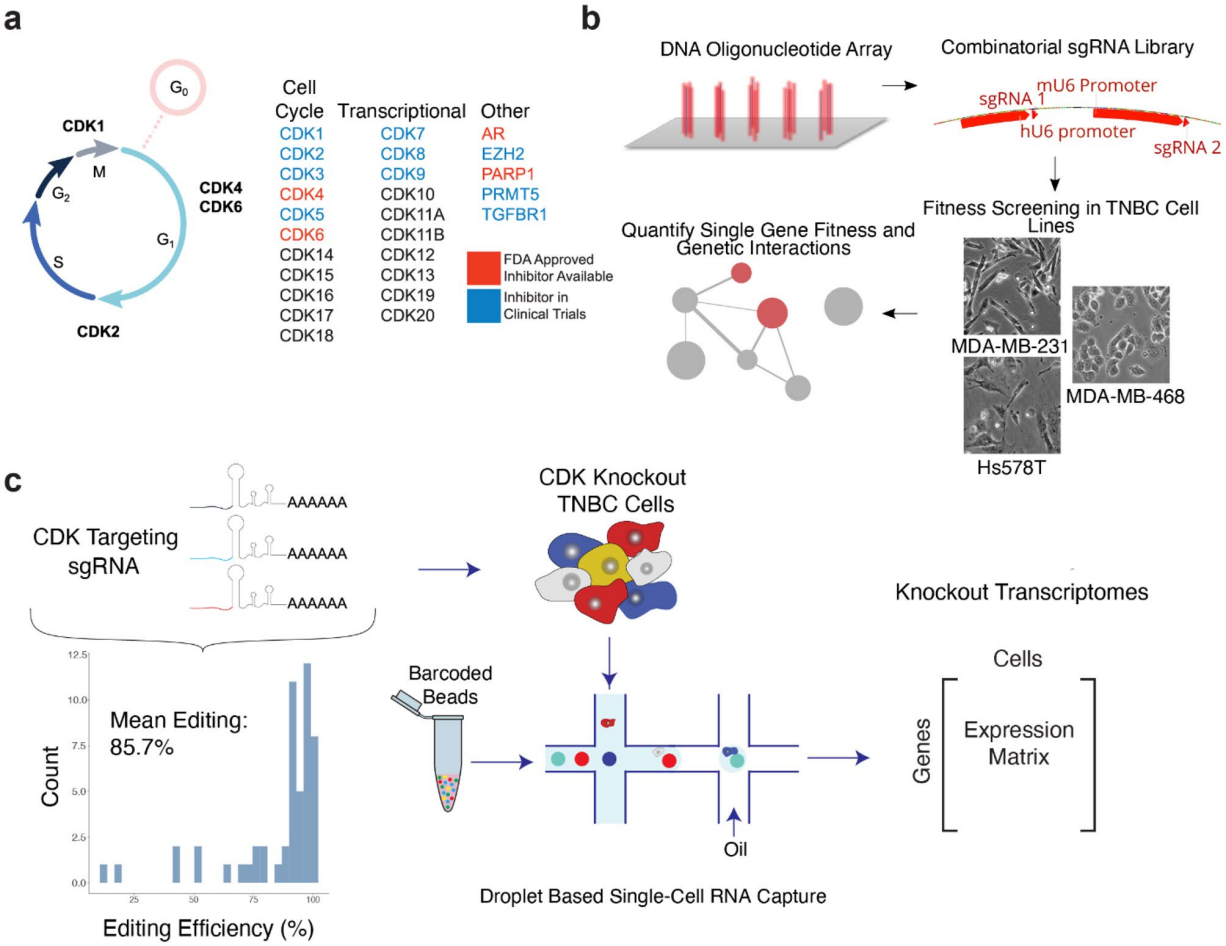
Systematic mapping of CDK gene function in triple negative breast cancer cells. (a) CDK proteins control cell-cycle progression and act as transcriptional regulators, garnering interest as potential drug targets (colors). (**b**) Schematic describing the combinatorial CRISPR/Cas9 fitness screening approach to map CDK synthetic-lethal and synergistic interactions. A library of dual sgRNA constructs targeting pairs of genes listed in (**a**) was synthesized as an oligonucleotide pool and cloned into a lentiviral overexpression vector (top). TNBC cell lines were transduced with virus coding for this library and subjected to competitive growth screening. Resulting dual sgRNA construct fitnesses were used to extract single gene fitness values and map genetic interactions. (**c**) Schematic describing the single-cell transcriptional phenotyping approach to map the functional impact of CDK genetic perturbations. An sgRNA library targeting the genes in (**a**) was cloned into an scRNA-seq-compatible lentiviral overexpression vector and used to transduce TNBC cell lines in pooled format. One week after transduction, scRNA-seq was performed using the 10x Chromium platform.

Regulation and transition between cell-cycle phases is accomplished primarily by cyclin-dependent kinases (CDKs) and associated cyclin proteins^1^. The CDK family is large, with more than 20 distinct protein-coding genes, and there is substantial uncertainty regarding the specific functions of individual family members^1,2^. Canonically, CDK proteins have been divided into two functional classes: factors that regulate cell cycle, such as CDK1, 2, 4 and 6, and factors that participate in general control of transcription, such as CDK7, 9 and 12^1^ (Fig. 1a, Extended Suppl. Fig. 1). The transcriptional CDKs play a critical role in regulating RNA Polymerase II (RNAPII), with diverse functions across initiation, elongation, and termination. CDK7,9 and 12 all have been shown to phosphorylate RNAPII directly. However, there is still much uncertainty regarding the mechanistic role and functional importance of each transcriptional CDK. For example, CDK8 (working as part of the Mediator complex) has been reported to be both a transcriptional repressor and activator, and CDK7 has established roles in initiation, capping, promoter-proximal pausing, and phosphorylation of CDK9^3,4^. CDK9 is essential for transcriptional elongation, with CDK12 knockdown also leading to global impairment in transcription, especially among long genes, and DNA damage response genes^1,5,6^. However, many CDKs have been shown to function in both cell-cycle and transcriptional roles as well as in diverse other pathways^7–16^. For example, both cell-cycle and transcriptional class proteins can activate the epigenetic regulators EZH2, AR, PRMT5, and PARP1^11,17–20^ or interact with proliferative cell signaling via the transforming growth factor beta (TGFβ) pathway^21,22^. The emerging picture is that CDKs govern a complex network of overlapping and synergistic functions, with “cell-cycle” and “transcriptional” labels providing useful but incomplete guidelines.

CDKs have also been the focus of extensive interest in the pharmaceutical industry, which has developed an armada of specific CDK inhibitors with potential applications in cancer^2,23^, infection^24,25^, neurological disorders^26–28^, and other diseases in which cell-cycle dysfunction plays a central role. Dual specificity CDK4/6 inhibitors have thus far shown tremendous benefit in cancer, with Phase III clinical trials for palbociclib reporting an improvement in progression-free survival of approximately ten months in combination with endocrine therapy in hormone-receptor positive (HR +) breast tumors^29^ (Fig. 1a). As these drugs have consequently moved to standard-of-care^2,30–33^, it has also become readily apparent that many tumors present innate or acquired resistance. One pathway to resistance is inactivation of the retinoblastoma tumor suppressor protein^34^ (Rb), a central transcriptional repressor of cell cycle progression which is regulated by CDKs. As Rb is typically inactivated in triple negative breast cancers (TNBC)^35^, CDK therapies have yet to be approved for this tumor subtype. Within the triple negative breast cancer classification, cells can be further divided into Basal A (more epithelial like), and Basal B (more mesenchymal). This stratification is the result of early gene expression profiling experiments^36,37^, which identified two distinct clusters of TNBC cells expressing genes similar to basal cells in the human mammary gland.

It is also clear that Rb status explains only a fraction of resistance to CDK4/6 inhibitors, motivating a keen interest in developing biomarkers of drug response^34,38^. For example, androgen receptor (AR) has been proposed as a biomarker for drug sensitivity^7^, and altered TGFβ signaling as a biomarker for drug resistance^39,40^. Another area of interest, particularly in TNBC, has been the identification of synthetic-lethal dependencies involving CDK proteins, i.e. protein pairs that selectively kill tumor cells when they are disrupted in pairwise combinations^38,41–43^. For example, inhibition of the epigenetic regulators EZH2 or PRMT5 is being investigated as a means to sensitize cells to anti-CDK4/6 therapy^12,44^, and inhibition of CDK12 was discovered to sensitize tumors to anti-PARP1 therapy^16,45,46^. Such developments suggest that the extended family of CDK proteins and interactors may provide a useful source of novel biomarkers and synthetic-lethal drug targets.

Here, we use CRISPR/Cas9 genetic disruption and single-cell mRNA sequencing^47–52^ to systematically interrogate interdependencies and functions of all 21 CDKs in TNBC cells, including 5 epigenetic factors linked to CDKs (AR, EZH2, PARP1, PRMT5, TGFBR1)^11,16–18,22^. These experiments reveal a complex network of synthetic-lethal interactions among CDKs and show that the cellular programs orchestrated by each CDK are remarkably diverse^49,53,54^. The resulting resource of interdependencies and associated cell states expands our understanding of this complex protein family and suggests targets for individual and combination therapy.

## Results

### A network of CDK genetic dependencies

To systematically map CDK genetic dependencies, we performed combinatorial CRISPR fitness screening using lentiviral vectors delivering pairs of sgRNA molecules to each cell^50^. We selected four distinct sgRNAs per gene, designed to perturb all single and pairwise combinations of the 26 CDK and CDK-related genes (Fig. 1a). Together with non-targeting sgRNA and safe-harbor controls (AAVS1, the adeno-associated virus integration site in intron 1 of PPP1R12C), this library design resulted in a total of 12,432 dual sgRNA constructs (Fig. 1b, “Methods”).

To supplement our combinatorial knockout screen with information-rich transcriptomic data, we built a second library of single-cell RNA sequencing (scRNA-seq) compatible single-knockout CRISPR constructs for the same set of 26 genes (2 sgRNA per gene). We verified the cutting efficiency of all 52 sgRNAs, confirming that we had achieved highly efficient editing of target loci (Fig. 1c). These libraries were used to interrogate three cell lines, representing distinct TNBC classifications (MDA-MB-468: Basal A; MDA-MB-231 and Hs578T: Basal B). MDA-MB-468 cells have a loss-of-function disruption of retinoblastoma protein (Rb–), while the Basal B cells are Rb + but have activating *RAS* mutations and *CDKN2A* deletions which increase mitogenic signaling via D-type cyclins^43,55–58^.

**Figure 2:**
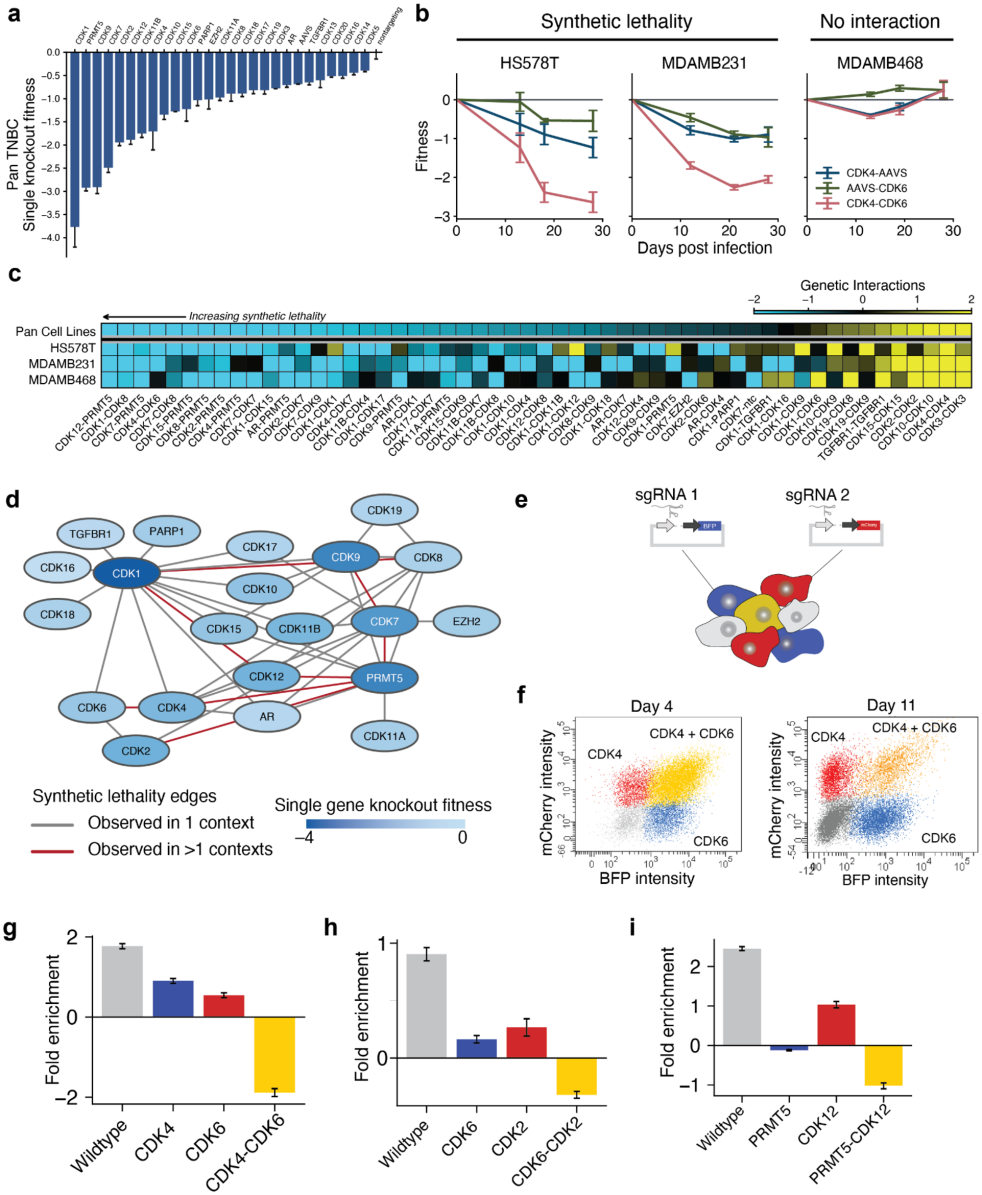
CDK combinatorial disruption reveals conserved and context-dependent interaction networks. (**a**) Mean fitness for cells receiving each CDK knockout, pooled across three TNBC cell lines. AAVS1, sgRNA targeting adeno-associated virus integration site 1, a safe-harbor control locus; NTC, non-targeting control. Error bars correspond to standard deviations across measurements from three cell lines: Hs578T, MDA-MB-231, and MDA-MB-468. (**b**) Fitness trajectories for *CDK4*/*6* dual knockout vs. single knockouts (pairing CDK4 or CDK6 with AAVS) in each TNBC cell background. Error bars correspond to standard deviation of fitness measurements across replicates and 32 guide pairs targeting the same gene pair. (**c**) Heatmap of significant genetic interactions for each cell line and a pan-cell line analysis. (**d)** Complete CDK synthetic lethality networks discovered across all experiments. Single gene knockout fitness is defined as the log_2_ growth relative to non-targeting control. (**e**) Schematic of validation of genetic interactions. sgRNAs paired with two different fluorophores are transduced at high MOI and grown in competition. Cells are colored according to the sgRNA a cell received: blue for sgRNA1-BFP, red for sgRNA2-mCherry, yellow for both sgRNA1-BFP and sgRNA2-mCherry, and gray for no viral integration. (**f)** CDK4/6 single and dual knockout populations 4 days and 11 days after infection. (**g–i**) Validation of synthetic lethal interactions for (**g**) CDK4-CDK6, (**h**) CDK2-CDK6, (**i**) CDK12-PRMT5 in MDA-MB-231 cells by fold enrichment (positive values) or depletion (negative values) of single and dual knockouts on day 11 vs. day 4 post infection. Error bars represent standard deviation across two replicates. Dual knockouts showed marked reduction in growth relative to single knockouts.

Cell lines were screened in biological duplicates, with genomic DNA sequenced at 4 time points over 28 days to track the relative fitness of cells harboring each dual sgRNA construct. Fitness measurements were well correlated between biological replicates (Pearson’s *r* = 0.996) and across the three breast cancer cell lines (*r* = 0.922 to 0.937), with *CDK1* ranking as the most deleterious knockout, consistent with its role as a master regulator of cell-cycle progression^32,59^ (Fig. 2a**, **Extended Suppl. Fig. 2). This high level of correlation was possible due to the large number (>100) of unique sgRNA constructs targeting each CDK gene and our computational strategy of imputing single gene fitness effects from the entirety of the combinatorial knockout data (“Methods”). We then analyzed these measurements to identify pairwise gene knockouts in which fitness was significantly less than or greater than expected from the single knockouts^50^ (Fig. 2b, “Methods”). This analysis identified a collection of 43 synthetic-sick/lethal and 12 synergistic genetic interactions, with CDK1-CDK12 identified as both synthetic-lethal and synergistic depending on context (Fig. 2c,d). These interactions were identified in either of two analysis modes: one treating data from each cell line separately, to identify specific vulnerabilities; another pooling all cell lines as replicates (“pan” cell line, Fig. 2c), to identify interactions occurring consistently across contexts with high statistical power.

Few synthetic lethalities identified in this experiment had been identified previously, with three partial exceptions. One interaction between CDK8 and CDK12 had been identified in K562, a model for chronic myeloid leukemia^52^. We saw this synthetic-lethal interaction in Hs578T, but not in the other two contexts. Two interactions, CDK4-CDK6 (Fig. 2b) and CDK2-CDK6 (Extended Suppl. Fig. 3a), had been previously inferred from patient data or knockout mouse experiments^60,61^ but not demonstrated with a combinatorial genetic screen. Here, we observed these interactions in our primary screen as well as an orthogonal flow cytometry assay (Fig. 2e–h, “Methods”). For the remaining novel synthetic lethals, 14 corresponded to protein pairs that had been shown to physically interact (Supplemental File 1), corroborating the observed genetic interactions.

Notably, genetic interdependencies among the canonical cell-cycle CDKs were observed exclusively in the Rb+ cell types (MDA-MB-231 and Hs578T). For example, strong synthetic lethality was observed between CDK4 and CDK6 in both of these backgrounds but not in the Rb– context (MDA-MB-468), supporting the use of Rb status as a predictive biomarker for efficacy of anti-CDK4/6 agents^42,62,63^ (Fig. 2b). We also observed Rb-dependent interaction of CDK2 with CDK6, of note due to ongoing research in trispecific CDK2/4/6 inhibitors^64^, as well as interaction of CDK1 with CDK17 and CDK18, suggesting that the Rb-dependent regulatory axis may include the broader family of cell-cycle CDKs beyond CDK2/4/6.

Other than the CDK4/6 dependency, many of the top five synthetic-lethal interactions featured a transcriptional CDK or epigenetic regulator (Fig. 2c*,* ranked by pooled score across cell lines). The overall strongest interaction linked PRMT5 and CDK12 (Fig. 2c,i; Extended Suppl. Fig. 3b), a novel interaction between two genes which, separately, have been implicated in regulation of RNA polymerase II (RNAP II) and splicing^16,65,66^. Related to this finding, we found synthetic lethalities linking PRMT5 to CDK7 and CDK9, two additional transcriptional CDKs (Extended Suppl. Fig. 3c,d). Several highly ranked synthetic-lethal interactions were identified linking a cell-cycle regulatory CDK to a transcriptional CDK, such as the CDK1–CDK8 interaction (Fig. 2d). Many synthetic lethalities involved CDK proteins that had yet to be investigated as anti-cancer drug targets, such as the transcriptional regulators CDK11B and CDK15.

### Effects of CDK knockouts on cell-cycle phase

Coupling genetic perturbations to rich molecular readouts, namely transcriptomic profiling with scRNA-seq^49^, offers the ability to reveal specific functions that underlie changes in fitness phenotypes. Accordingly, we analyzed each of the three TNBC cell lines using scRNA-seq in the presence or absence of genetic disruptions to each of the 26 CDK and CDK-related genes (Fig. 1c). A pooled library of CRISPR single-guide RNAs (sgRNAs) was transduced at low multiplicity of infection (MOI) such that the majority of cells received at most a single sgRNA (Extended Suppl. Fig. 4). One week after transduction, scRNA-seq was performed using the 10x Chromium platform (“Methods”). When annotating which cells received which sgRNA, we observed fewer than expected (based on the equimolar starting pool of CDK targeting sgRNA) cells harboring sgRNAs targeting essential genes such as CDK1 (Extended Suppl. Fig. 4c), consistent with their negative effects on cell fitness.

Within these data, we examined the expression of 603 genes that had been previously nominated as cell-cycle markers based on their periodic transcriptional variation in cycling cells^67–69^. Gene markers of the same cell-cycle phase were tightly clustered when examining their co-expression (Pearson correlation, “Methods”), supporting their previous assignments (Extended Suppl. Fig. 5a). Furthermore, these clusters included additional transcripts whose inclusion was consistent across the three cell lines, prompting us to expand the set of cell-cycle markers by an additional 127 genes (Extended Suppl. Fig. 5b–d, “Methods”). We found highly significant overlap between this expanded list of cell-cycle marker transcripts and an independent dataset of cell-cycle transcripts characterized by the Human Protein Atlas^68^ (p = 1.64 × 10^–31^ Fisher's exact test, odds ratio = 49.5; Extended Suppl. Fig. 5c). There was less overlap between our expanded list of cell-cycle marker transcripts and known cycling proteins, likely due to the importance of post-translational mechanisms in regulating cell phenotypes at the protein level^70^ (Extended Suppl. Fig. 5c). Of the 127 additional cell-cycle markers, 34 were differentially expressed in one or more CDK knockout populations (Extended Suppl. Fig. 5e).

**Figure 3:**
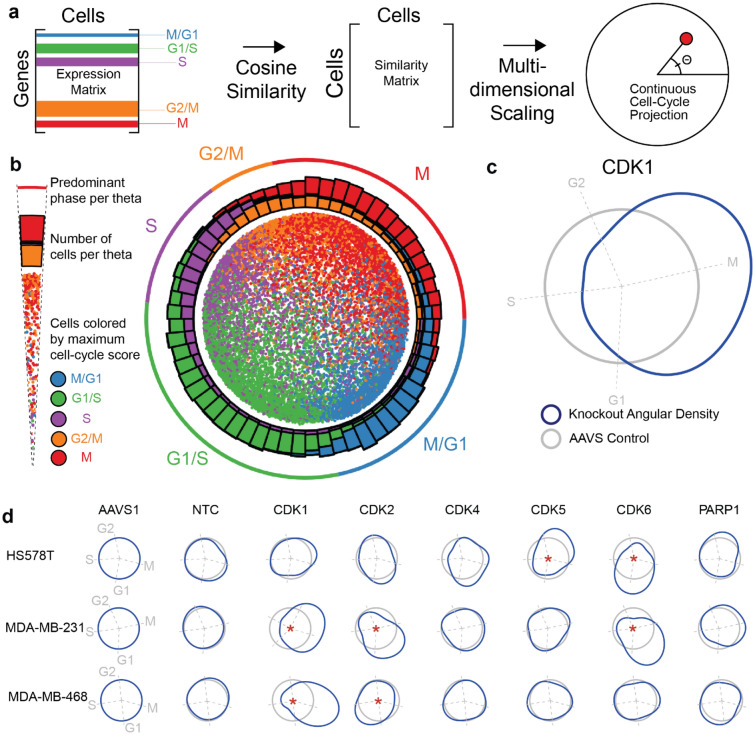
Effects of CDK disruption on cell-cycle phase. (**a**) Approach for embedding cells such that cell-cycle phases can be measured. In the embedding, the angle Θ indicates phase. (**b**) Cell-cycle embedding of all MDA-MB-231 cells. (**c**) Deviation of CDK1 knockout cells from AAVS control cells (grey circle) in density of cells about the cell-cycle embedding (blue). Dashed lines represent the median angle of cell-cycle phases. (**d**) Deviation in single-cell density compared to AAVS for select knockouts in MDA-MB-231, Hs578T, and MDA-MB-468 cells; *p < 0.05 by Kuiper test.

The cell-cycle phase of each cell was determined by embedding the expression profiles of the expanded set of cell-cycle markers into polar coordinates, similar to a previous method based on Hi-C data^71^ (Fig. 3a, “Methods”). In these coordinates, angle corresponded to the state of cell-cycle progression at the time of cell capture, with M, G1, S and G2 phases defined by successive angular ranges around the unit circle (Fig. 3b**, **Extended Suppl. Fig. 6a,b). The subpopulation of cells harboring a specific CDK knockout could then be selected, and its angular distribution examined for aberrations relative to wild type (Fig. 3c). Using this approach, we found that knockouts of CDK1, 2, 5, and 6 all had significant effects on cell cycle progression (Fig. 3d). Cells harboring CDK1 knockouts accumulated at the end of G2 phase, whereas cells harboring CDK2 knockouts accumulated at G1^72^ (Fig. 3d). CDK2 and CDK5 had context-specific impacts on cell cycle: CDK2 knockouts resulted in M/G1 arrest in the Rb+ lines and early S phase arrest in the Rb– line, while CDK5 knockouts arrested in G2/M only in Hs578T cells. The effects of CDK6 knockout were also context-dependent: MDA-MB-231 and Hs578T cells showed enrichment in early and late G1 respectively, whereas the Rb– line, MDA-MB-468, showed little cell-cycle effect. In addition to effects of these canonical cell-cycle CDKs, we found that CDK13 significantly perturbed cell cycle progression in Hs578T cells, although it has previously been classified as transcriptional CDKs (Extended Suppl. Fig. 6c). We further used the angular cell-cycle embedding to robustly remove cell-cycle signatures from the expression profiles (Extended Suppl. Fig. 6f).

### CDK transcriptional effects are large and distinct from one another

**Figure 4:**
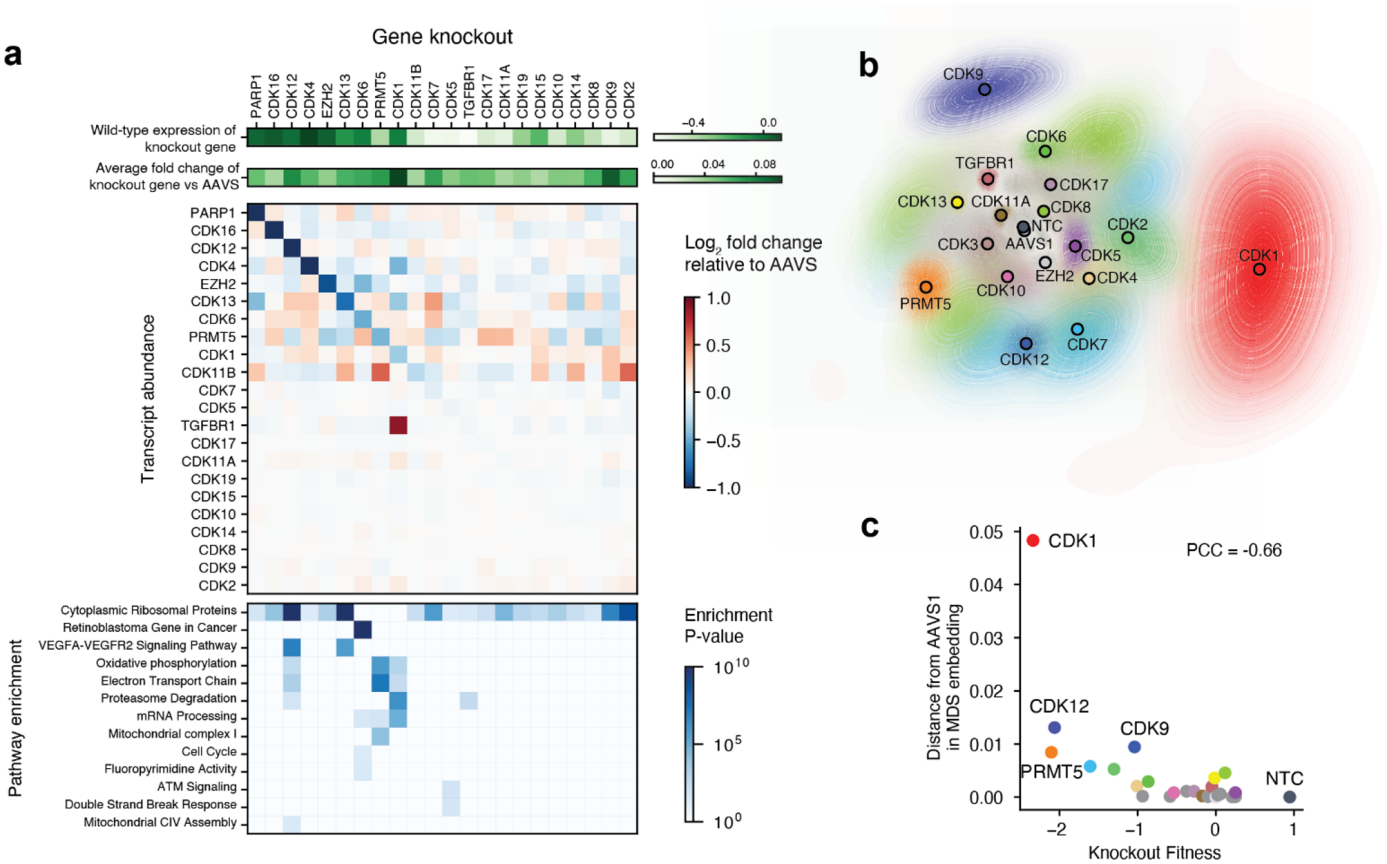
Effects of CDK disruption on diverse transcriptional programs. **(a**) Wild-type expression (top row) of CDK genes (columns) and the knockout effect of those genes on their own expression (second row), the expression of other CDK genes (third row), and specific pathway signatures (bottom row) in MDA-MB-231 cells. (**b**) MDS embedding of median single cell profile for each gene knockout. Each contour line depicts the confidence interval across 1000 bootstrap resamplings. The outermost contour line represents the 95% confidence interval. (**c**) For each gene knockout (colored points), the distance of the transcriptome from the AAVS control (y-axis) is plotted versus its fitness.

We next sought to quantify the functional effects of CDK knockouts beyond cell-cycle progression. We chose to focus our analysis on the MDA-MB-231 cell dataset, due to it having the highest number of cells harboring single sgRNA (increasing statistical power). First, we confirmed that many of the knockouts led to a significant expression reduction of the corresponding gene *in cis*, consistent with nonsense-mediated decay of the CRISPR-edited transcripts^73^. CDKs lacking this *cis* regulatory effect could be largely explained by low endogenous transcript abundance levels in wild-type cells (Fig. 4a), as CRISPR sgRNA reagents were confirmed to efficiently generate gene knockouts (85.7% mean editing rate, Fig. 1c).

Moving to *trans*-acting effects, we found that many CDKs have strong transcriptional effects that are very different from one another in the affected downstream genes and pathways (Fig. 4a,b**, **“Methods”). In particular, CDK1 knockout in MDA-MB-231 cells showed significantly perturbed expression of a large number of genes (1334), including the TGFβ receptor (TGFBR1) as well as genes involved in proteasomal degradation, oxidative phosphorylation and the electron transport chain (Fig. 4a). CDK5 knockouts showed perturbed transcription of DNA damage response genes, potentially due to the observed dysregulation of DNA damage signaling via ataxia-telangiectasia mutated (ATM)^74^. While CDK6 knockouts caused dysregulation of Rb-regulated genes and canonical cell-cycle genes, they additionally perturbed genes involved in metabolism of fluoropyrimidines. The classic transcriptional CDKs also impacted diverse pathways. While CDK7, CDK9, and CDK12 knockouts each had highly perturbed transcriptomes when compared to control cells (in MDA-MB-231 cells; 92, 347, 893 differentially expressed genes, respectively, p_adj_ < 0.05; Fig. 4b,c), we detected few commonly dysregulated cell functions save for VEGFA-VEGFR2 signaling in CDK12 and CDK13 knockouts (Fig. 4a). Regardless of these differences, the magnitude of transcriptional perturbation caused by a CDK knockout (Fig. 4b, Extended Suppl. Fig. 7a,b, radial distance from AAVS control) was strongly and negatively correlated with its effect on cell fitness (Fig. 4c, Pearson’s r = –0.66, Extended Suppl. Fig. 7a,b). Thus, transcriptional effects of CDK knockouts scale with their effects on growth, but beyond this general association they implicate different underlying programs.

### The CDK/RNAPII transcriptional axis presents a critical vulnerability in TNBC cells

**Figure 5:**
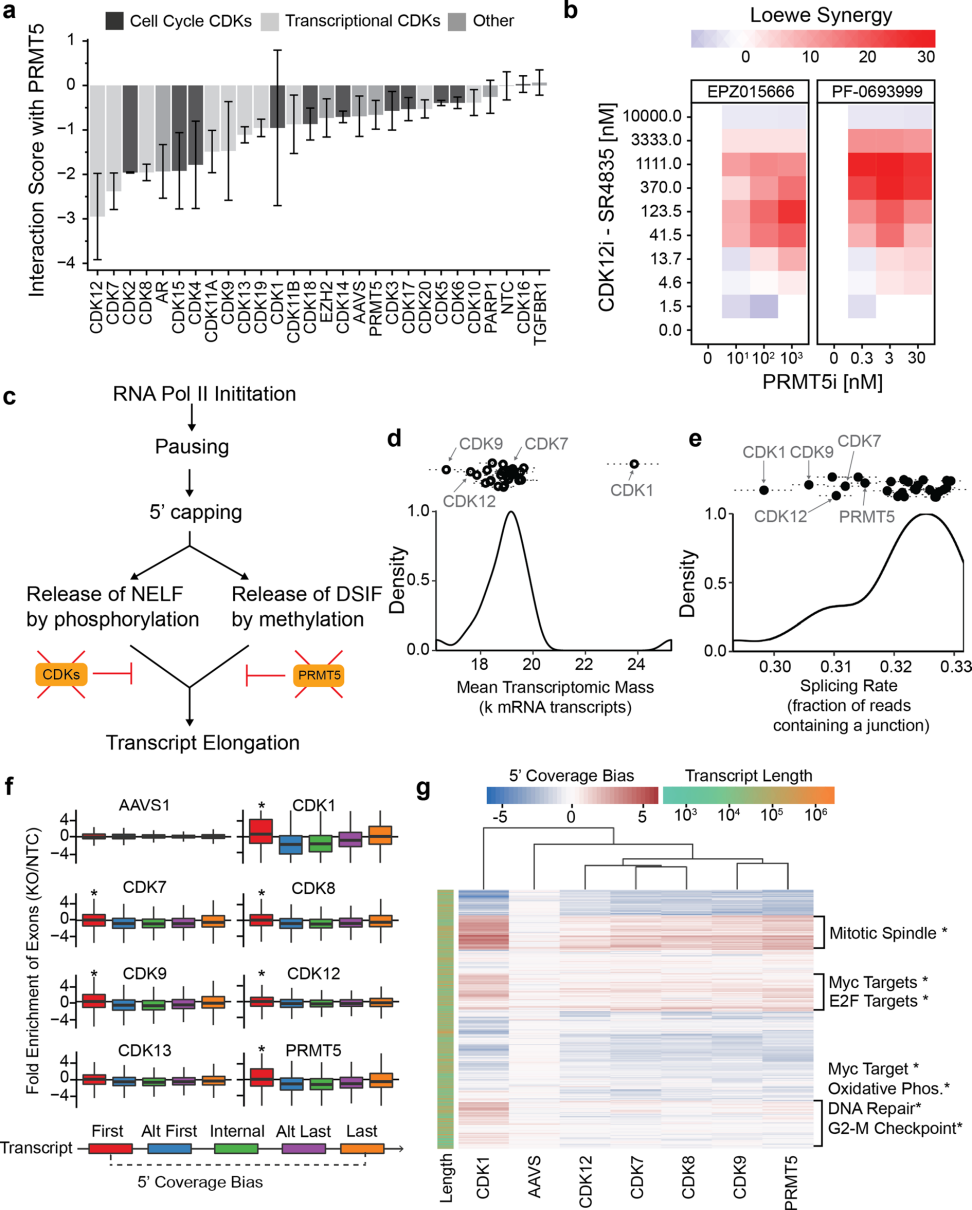
Relation of PRMT5/CDK synthetic-lethal interactions to aberrant splicing. (**a**) Genetic interaction score of indicated gene in combination with PRMT5, pooling data from MDA-MB-231, Hs578T, and MDA-MB-468 cell lines as replicates. Error bars represent the standard deviation across all replicates and cell lines. (**b**) Synergistic inhibition of MDA-MB-231 cell growth with combinatorial treatment of a CDK12 inhibitor (SR-4835) and a PRMT5 inhibitor (EPZ015666 or PF-0693999). (**c**) CDK proteins and PRMT5 modulate transcript elongation. (**d**) Mean number of transcripts observed in cells impacted by each gene knockout. The dotted lines represent the standard error of the mean. (**e**) Splicing rate observed across single cells impacted by each gene knockout. Dotted lines span the standard error of the mean. (**f**) Log_2_-fold coverage of exon positions (colors) in transcripts from cells harboring specific gene knockouts (subplots). Data are normalized against data from cells harboring non-targeting-control guides (*p < 0.05, t-test compared to AAVS). (**g**) Heatmap showing the 5′ coverage bias (first exon relative to last exon) for each gene (row) under select gene knockouts (columns). The most enriched biological functions (MSigDB Hallmark gene sets) are given for select clusters of genes (*p_adj_ < 0.05). Rows and columns are sorted by hierarchical clustering; the dendrogram of rows is not shown. Data in panels (**d–g**) are from MDA-MB-231 cells.

Our genetic interaction analysis revealed that three of the classical transcriptional CDKs (CDK7, 9, 12) have strong synthetic-lethal interactions with the transcriptional regulator PRMT5 in all three cell-line contexts, with the CDK12-PRMT5 interaction being the strongest in the screen overall (Figs. 2c, 5a). We further confirmed this interaction in two ways: first using an independent FACS assay (Fig. 2h), and second using selective small molecule inhibitors against CDK12 (SR4835) and PRMT5 (EPZ015666 or PF-06939999) in place of CRISPR guides (Fig. 5b).

Phosphorylation of the carboxy-terminal domain (CTD) of RNA polymerase II (RNAPII) by CDK7, CDK9, and CDK12 is crucial for release of the negative elongation factor (NELF), promoting transcription^5,75,76^. Likewise, methylation of SPT5 by PRMT5 dissociates the DSIF repressor from RNAPII^65^, thus promoting transcript processing. Given these convergent functional roles (Fig. 5c), we examined how CDK7/9/12 and PRMT5 functions impact RNA production and splicing patterns across the transcriptome. First, we found that the expression levels of an NELF subcomponent, NELFE, were significantly dysregulated in CDK9/12 and PRMT5 knockout cells (p < 0.05 t-test; log_2_ fold-changes of 0.24, -0.86, -0.23, respectively; Extended Suppl. Fig. 8a,b). In addition to NELFE, several other key RNAPII associated proteins had changed expression levels in response to CDK knockouts, including RNAPII subunits in all CDK knockout populations. Second, we noted that CDK9 and CDK12 knockouts produced very low transcriptional activity (read count per cell, Fig. 5d), as would be expected given the similar role of these kinases in NELF release by phosphorylation of the RNAPII CTD at Ser-2^77^ (Fig. 5c). Notably, CDK7 knockouts did not show marked reduction in transcriptional activity by this metric, in contrast to prior work implicating CDK7 in transcriptional initiation via the TFIIH complex and in transcriptional elongation via CDK9 phosphorylation^4,78,79^. However, our data supported previous research showing CDK7 is not essential for global transcription^80,81^, highlighting that although CDK7/9/12 all converge on RNAPII, the kinases have unique functional roles (and differing levels of essentiality) in RNAPII regulation. Showing a remarkably different trend, CDK1 knockout cells had greater transcriptional activity, although we were unable to mechanistically deconvolve this result from CDK1 mediated cell-cycle regulation.

Third, we found that knockouts of CDK7/9/12, as well as PRMT5 and CDK1, led to a reduced fraction of spliced transcripts across the transcriptome (Fig. 5e), highlighting that although CDK7 knockout did not markedly reduce overall transcriptional activity, it did quantifiably perturb the fidelity of transcription. Fourth, in addition to a reduction in splicing overall, CDK7, 9 and 12 knockouts led to transcripts with significantly increased representation of the first exon, and significantly decreased representation of subsequent exons, relative to wild-type cells (p < 0.05 t-test; Fig. 5f). PRMT5 and CDK1 knockouts also led to significantly increased representation of the first exon, indicating perturbed transcription. Thus, an in-depth analysis of mRNA expression and splicing patterns provides multiple lines of evidence that the CDK-PRMT5 dependency is due to aberrant transcriptional activity resulting in a reduction in full-length processed mRNAs. However, the co-regulatory nature of the transcriptional CDKs, such as CDK7 phosphorylating CDK9, as well as the diverse sets of genes that become differentially expressed upon targeted knockout, allow for the possibility that other unidentified proteins may be critical for mediating the observed transcriptional changes.

To further characterize the impact CDKs and/or PRMT5 inhibition have on RNA Pol II transcription, we leveraged a CUT&Tag^82^ assay to profile RNA Pol II activity across the genome in individual CDK knockdowns, as well as in combination with PRMT5 knockdown (Extended Suppl. Fig. 9). Using an antibody raised against a synthetic “YSPTSpPS” peptide corresponding to the Ser-5 phosphorylated RNAPII C-terminal domain, we profiled direct interactions between phosphorylated RNAPII and the genome, more directly assaying transcriptional initiation/activity than had our scRNA-seq readout. The results of this CUT&Tag assay demonstrated that CDK7, CDK12, and PRMT5 single knockdowns experience a significant reduction in RNA Pol II signal near the transcriptional start site compared to the NTC controls, and all of the combination knockdowns showed this transcriptional defect. This transcriptional phenotype supports previous work to selectively inhibit CDK7 and CDK12 with ATP analog inhibitors^5,83^, highlighting that while CDK7 is often considered the primary regulatory CDK for transcriptional initiation, there is extensive CDK cross-talk during this process. We also observed reduced RNA Pol II signal near the TSS for PRMT5 knockdown cells, providing new evidence for how PRMT5 regulates transcription beyond its more established functional role in splicing^65^.

Following this observation, we next sought to determine the particular groups of genes for which splicing and other transcriptional dysregulation were most affected. For this purpose, we quantified the “5’ coverage bias” of a gene as the relative abundance of the first exon relative to the last exon among the collection of all transcript isoforms identified for a gene (Fig. 5f). When looking across the entire transcriptome, we observed that very similar sets of genes had high 5’ coverage bias in response to knockout of CDK7, 9, 12 or PRMT5 (Fig. 5g). Moreover, these groups of genes were significantly enriched for key cellular functions, including mitotic spindle formation and DNA repair (p_adj_ < 0.01, odds ratios of 3.97 and 5.05, respectively; Fig. 5g). Notably, a strong 5’ coverage bias was observed among targets of the central transcriptional activators MYC and E2F (p_adj_ < 0.01, odds ratios of 3.92 and 5.35, respectively; Fig. 5g), suggesting that dependence of TNBC on complete transcription of MYC and/or E2F targets may underlie the observed CDK/PRMT5 synthetic lethality.

## Discussion

Integrating complementary pooled screening methodologies has the potential to substantially improve our understanding of genotype–phenotype relationships, including those in disease. Because CRISPR-Cas9 perturbs CDK function by specific disruption of genomic DNA, it bypasses confounding issues seen with chemical perturbagens such as off-target effects (given that CDKs have high sequence homology to one another) and the inability to inhibit phosp27-CDK4-CycD1 complexes^84,85^. While we focused on CDK proteins, similar approaches can be applied to diverse other biological pathways of interest. For example, combinatorial transcription factor expression is critical for cellular differentiation and development^86^ and could be readily assayed in a similar fashion via CRISPR reagents and scRNA-seq. Additionally, the framework established here for visualizing the cell-cycle phenotypes of individual cells in scRNA-seq data could be applied to alternative phenotypes defined by sets of genes.

The 43 synthetic-lethal interactions we identified among CDK genes precisely quantify the functional redundancies and interdependencies that exist in this gene family. While early studies of CDK4 and CDK6 suggested they were functionally redundant^60^, our results highlight distinct roles based on several lines of evidence. First, each of the single CDK4 and CDK6 knockouts has a negative fitness impact, meaning its function is not completely buffered by the other gene (Fig. 2a). Second, knockouts of CDK6, but not CDK4, significantly alter cell-cycle progression (Fig. 3d). Third, only CDK6 knockouts result in significant deregulation of Rb controlled genes (Fig. 4a). Fourth, CDK4 has many more synthetic-sick/lethal interactions than CDK6 (7 versus 3, Fig. 2c,d). One explanation for these distinct effects is that CDK4 is more readily compensated by diverse members of the CDK family. On the other hand, in support of some redundancy, CDK4 and CDK6 knockouts are synthetic-sick/lethal with each other (Fig. 2d–g). This redundancy likely relates to their shared regulation of the Cyclin-D/Rb signaling axis, given the lack of CDK4/CDK6 synthetic lethality in Rb– cell lines^87^ (Fig. 2c).

Contrary to the usual stratification of CDK genes into “cell-cycle” or “transcriptional” families (Extended Suppl. Fig. 1), each with independent functions, here we observe many genetic dependencies across CDKs of these two classes (Fig. 2d). This crosstalk is reflected in the transcriptome as well, where single-cell RNA sequencing reveals extensive transcriptional regulation by CDK1, a canonical cell-cycle regulator (although deconvolving transcriptional changes due to impaired cell fitness from regulatory activity is an ongoing challenge). Furthermore, we find that cell-cycle regulation is far from uniformly conserved across cellular contexts, since the same gene knockout (e.g. CDK2, 5, 6) can have impacts on cell-cycle behavior that are largely distinct from one another depending on the cell line (Fig. 3d). These results suggest that the exact timing, mechanisms, and druggability of cell-cycle checkpoints are not universal^88,89^.

Our analysis also indicates that the previously underexplored CDK7, CDK9, and CDK12 proteins play critical roles in controlling cell proliferation and RNAPII activity in concert with PRMT5 (Fig. 5). We observe a synthetic lethal phenotype when CDK7, CDK9 or CDK12 are knocked out in combination with the RNAPII regulator PRMT5, supporting emerging research that sequential phosphorylation of RNAPII by multiple CDKs (CDK9 and CDK12 phosphorylate Ser-2 on the RNAPII CTD, while CDK7 phosphorylates Ser-5) is critical for proper RNAPII function^77^. Unlike CDK9 and CDK12, knockout of CDK7 does not result in a global reduction of detected transcripts (Fig. 5d), suggesting that phosphorylation at RNAPII CTD Ser-2 is the more critical regulatory event for RNAPII function. Regulation of transcriptional activity via the combination of these proteins emerges as a critical fitness vulnerability, with promising avenues for drug development and therapeutic intervention. Our observation that CDK7, 9, 12 and PRMT5 knockouts have improper transcription of MYC-regulated transcripts is especially important, given that MYC is an amplified oncogene in the majority of TNBCs^90^. These results suggest that other regulators of transcriptional activity and splicing outside the CDK family might serve as potential drug targets as well^91^. In support of this notion, PRMT5 inhibition has been shown to be synergistic with inhibition of DOT1L, a methyltransferase that regulates RNAPII^92^. CDK13 mutations have recently been shown to drive melanoma growth via ZC3H14-regulated improper transcriptional elongation, suggesting that the fitness impact of transcriptional dysregulation depends specifically on which transcripts are being perturbed^93^. Additional studies will be able to assess the potential effects of therapeutically targeting the various steps of transcription (initiation, elongation, termination) on diseased and healthy cells in vivo^94^.

While these results expand our understanding of CDK function and essentiality in cell-cycle transition and transcription, there are still mechanistic uncertainties yet to be understood. One challenge encountered in this study is the difficulty in interrogating essential genes. Knocking out essential kinases, such as CDK1, results in a massive loss of fitness, severely reducing cell numbers available for transcriptional profiling in a pooled screen (Extended Suppl. Fig. 4c). One potential solution to this problem is to pool CRISPR sgRNAs predicted to cause large fitness defects at higher abundance in the initial library construction. Another challenge in understanding CDKs via scRNA-seq is the discrepancy between protein levels and RNA levels. Some cell-cycle proteins are regulated post-translationally^68^, limiting their usefulness in assaying the cell cycle when using a transcriptional readout. Given the importance of proteins in mediating biological phenotypes, advances in single-cell (and other high-throughput) proteomics will surely expand the potential toolkit for screening gene/protein function.

Here, we have presented a systematic, unbiased resource of CDK functions and interdependencies governing cellular growth, cell cycle, and transcriptional programs. Perturbations to essential cell functions such as transcription yield major impacts to cell state, with quantifiable effects unique to each CDK protein. Given the fundamental roles that CDK signaling plays in disease etiology and treatment, this dataset has the capacity to inform both basic science and translational medicine. We anticipate that our quantitative mapping of CDK gene functions will guide future interrogations into CDK biology, helping uncover how this critical class of proteins can be further leveraged therapeutically.

## Methods

### Phylogenetic tree construction

Tree diagram showing relationships between CDK proteins was constructed from a multi-sequence alignment (MSA) using Geneious^95^. The “Geneious Aligner”, was used to generate the MSA, and the neighbor joining method was used to construct the tree. All default parameters were used except where otherwise indicated.

### Combinatorial CRISPR sgRNA library construction

#### Design of gRNA spacer sequences

A list of 21 CDK and 5 non-CDK genes was compiled from literature sources. The HGNC symbols of these genes were converted to Entrez IDs using Bioconductor packages AnnotationDbi and org.Hg.eg.db. To target these genes in CRISPR-Cas9 knockout experiments, four different gRNA spacer sequences were selected per gene from two lists of such sequences. One list was obtained from the Genetic Perturbation Platform sgRNA Designer (GPPD) web tool (https://portals.broadinstitute.org/gpp/public/analysis-tools/sgrna- design, accessed in March 2018), and the other from the Brunello lentiviral pooled library (https://www.addgene.org/pooled-library/broadgpp-human-knockout-brunello/). The latter consists of 76,441 validated gRNAs that target 19,114 human genes and includes 1,000 control gRNAs^96^. To obtain the first list of gRNA spacer sequences, the Entrez IDs of the target genes were submitted to GPPD with the following parameters: enzyme = Sp, taxon = human, quota = 50, include = unpicked. The output of this tool was a table listing up to 50 candidate spacers for each specified gene. For each spacer, the table included the genomic location (chromosome, coordinate, and strand) of the cut site, the 20-nt target sequence, a 30-nt context sequence encompassing the cut site, the PAM sequence, and the “pick order”, i.e. the gRNA ranking order based on a score that combines predictions of on-target and off-target Cas9 activity^97^. To detect potential errors, the obtained spacer sequences were subjected to the following quality control steps. The initial list of 6349 sequences was searched for duplicate entries, 330 of which were found and discarded. For each of the remaining 6019 spacers, a 30-nt context sequence around the cut genomic location predicted by GPPD was extracted from the human genome assembly hg38 using Bioconductor package BSgenome.Hsapiens.UCSC.hg38. The extracted sequence was compared to the 30-nt context sequence reported by GPPD. An exact match between the two sequences was found for all of the tested spacers. Next, each spacer sequence was tested for targeting the intended gene. To this end, the annotation file gencode.v28.annotation.gtf.gz was downloaded from release 28 of the GENCODE project, and a list of coding sequence (CDS) annotations for the human genome was extracted from that file. All gene IDs in the list of spacers were found to be represented in the extracted list of CDSs. Each spacer was tested to verify that the predicted genomic location of the cut site was within the annotated CDSs of the target gene, and not within the CDSs of any other gene. A suitable CDS could not be found for 11 spacers, but these had not been picked by GPPD and were therefore discarded at a later stage (see below). Lastly, to test for potential off-target activity, the spacer sequences were mapped to the human reference genome using Bioconductor packages Biostrings and BSgenome.Hsapiens.UCSC.hg38, allowing for up to two base mismatches. Out of 6019 sequences, 3697 mapped to multiple genomic locations. In the latter group, 43 spacers were found to have a pick order less than 5. The second list of spacer sequences was obtained by downloading the file https://www.addgene.org/static/cms/filer_public/8b/4c/8b4c89d9-eac1-44b2-bb2f-8fea95672705/broadgpp-brunello-library-contents.txt. The table in this file contained the same kind of information as that provided by GPPD. This table was confirmed to contain no two spacers with the same predicted cut site, or with the same target sequence, or with different lengths of target, context, or PAM sequence. The list of spacers was then subjected to the same quality controls described above for the list of spacers obtained from GPPD. In this case, 784 spacers were found to be associated with 196 genes lacking a CDS annotation, 48 spacers did not hit a CDS of the intended gene, 790 spacers hit a CDS of 211 genes that were not the intended targets, 12 spacers hit only the CDSs of unintended targets, and 74,831 spacers hit only a CDS of the intended targets. Within this last set of spacers, 30,481 could be mapped to multiple genomic locations with up to two base mismatches. All CDS hits were determined using the downloaded and confirmed genomic locations of the gRNA cut sites. After the above controls, the two lists of spacers obtained from GPPD and the Brunello library were merged into a single list. All spacers labeled with the Entrez IDs of the 26 chosen genes were retained, yielding 6,024 spacers. From the latter set of spacers, a total of 5236 undesirable spacers were discarded. These included 11 spacers that were not hitting a CDS of the intended gene, 4745 that were not assigned a pick order by GPPD, and 2647 whose target CDS was not one of the following: the only CDS in the gene, the second CDS in the gene, or an “asymmetric” exon, i.e., a CDS that is not the first or the last in the gene and whose length in bases is not a multiple of 3. These criteria for choosing the target CDS were intended to maximize the likelihood of disrupting the translation product from the targeted gene. Out of the remaining spacers, 104 were selected to target the 26 chosen genes, with four spacers per gene. To make this selection, the spacers in the Brunello library were given the highest priority, and the genes obtained from GPPD were ranked according to pick order. The final list of selected spacers (Supplemental File 1) included 60 from the Brunello library and 44 from GPPD. This list of 104 gene-targeting spacer sequences was augmented with four non-targeting sequences (AAAAAGCTTCCGCCTGATGG, AACTAGCCCGAGCAGCTTCG, AAGTGACGGTGTCATGCGGG, AATATTTGGCTCGGCTGCGC) and four sequences targeting the AAVS1 safe harbor locus (CCTGCAACAGATCTTTGATG, GGTCCAAACTTAGGGATGTG, AGTACAGTTGGGAAACAACT, GGCCATTCCCGGCCTCCCTG). The final list was used to generate a pool of oligonucleotide sequences containing all possible pairs of spacer sequences, but excluding pairs of identical sequences, thus yielding (104 + 8) × (104 + 8 − 1) = 12,432 different pairs. For each such pair, the corresponding oligonucleotide sequence was obtained from the following scaffold sequence:

TCTTGTGGAAAGGACGAAACACCG <M20> GTTTTGAGACG <R15> CGTCTCGTTTG <N20> GTTTTAGAGCTAGAAATAGCAAGTTAAAA, where the segments <M20> and <N20> were replaced with the given pair of spacer sequences, and the segment <R15> was replaced with a unique random 15-base sequence. The latter was intended to minimize the “uncoupling” of spacer sequences that can arise from abortive PCR products^98^. To obtain the random 15-base sequences, a pool of 592 barcodes of length 5 bases and minimum Hamming distance of 3 bases was generated using the function DNABarcodes in the Bioconductor package of the same name^99^. This function was used with the parameter heuristic = "ashlock". A unique permutation of three 5-base barcode sequences was used to define each of the 15-base random sequences. The list of oligonucleotide sequences was submitted to CustomArray, Inc. (Bothell, WA) for synthesis on CMOS array technology.

#### PCR amplification of pooled oligos

The dual library constructs were ordered as single stranded DNA oligonucleotides from Custom Array. PCR primers OLS_gRNA-SP_F and OLS_gRNA-SP_R were used to amplify 100 ng of the libraries with Kapa Hifi Hot Start Ready Mix (Roche 7958935001) according to the manufacturer's protocol. An annealing temperature of 55 °C and an extension time of 15 s was used, with the number of cycles tested to fall within the exponential phase of amplification.

#### Gibson cloning of amplified libraries into lentiviral plasmids

A lentiviral vector containing Cas9 and a human U6 promoter for sgRNA expression (LentiCRISPRv2: Addgene 52961) was digested with BsmBI (NEB R0580) for 3 h at 55 °C. The digested vector was then purified using a Qiaquick PCR purification column (Qiagen 28104). Gibson Assembly reactions containing 200 ng of digested vector, 36 ng of insert (containing pooled library), and 10 μL of Gibson Assembly Master Mix (NEB E2611S) were then incubated at 50 °C for 1 h, and subsequently transformed into 200μL of Stbl4 electrocompetent bacteria (Thermo 11635018). Transformed cells were resuspended in 8 mL of SOC media (Invitrogen 15544034) and allowed to recover for 1 h shaking before being used to inoculate 150 mL of LB media supplemented with carbenicillin. After 16 h of further growth, plasmid DNA containing the sgRNA library was isolated via a Qiagen Plasmid Plus MaxiPrep kit (Qiagen 12963).

#### Insertion of the gRNA scaffold, mouse U6 promoter, and 30mer barcode

A DNA insert containing the mouse U6 promoter and second gRNA scaffold was first PCR amplified from a previously sequence validated TOPO vector (Shen et al.^50^). This insert was modified from previous designs to include a 30mer Unique molecular identifiers (UMI) barcode between each pair of sgRNAs. To generate this modified insert, 5’ and 3’ fragments of the original insert were amplified using dgRNA_Insertv4_barcoded_Left_F/R and dgRNA_Insertv4_barcoded30mer_Right_F/R, respectively. These two fragments were then stitched together via an overlap extension PCR and subsequently cloned into the sgRNA library containing vector. 10 ng of template plasmid was used to amplify the 5′ and 3′ fragments, with an annealing temperature of 65 °C and an extension time of 30 s and 25 cycles. After purifying via a Qiaquick PCR Purification column, the two fragments were stitched together via an overlap extension PCR amplification using primers dgRNA_Insertv4_barcoded_Left_F and dgRNA_Insertv4_barcoded_Right_R, with identical PCR cycling conditions as the individual fragment amplifications. 147 ng of the purified 3′ fragment and 52 ng of purified 5′ fragment were used as template to maintain an equimolar concentration of each fragment.

#### Insert ligation and transformation

Both the insert and step 1 sgRNA vector were digested with BsmBI for 3 h at 55 °C and subsequently purified via a Qiaquick PCR Purification column. The ligation reactions were then set up using 100 ng of vector, 100 ng of insert, 2 μL of buffer, 1 μL of T4 ligase (NEB M0202T), and ultra pure H_2_O up to 20 μL. Each reaction was allowed to proceed overnight at 16 °C. The following morning the ligase was heat inactivated at 65 °C for 20 min. Following this, the reaction was dialyzed into ultrapure water (Millipore VSWP01300) to remove any residual salts from the ligase buffer. Once the DNA was dialyzed, the ligation reaction was split evenly between 300 μL of Stbl4 electrocompetent cells, which were then transformed according to the manufacturer's protocol. The transformed cells were resuspended in 10 mL of SOC media (Invitrogen 15544034) and allowed to recover for 1 h shaking before being used to inoculate 150 mL of LB media supplemented with carbenicillin. After 16 h of further growth, plasmid DNA containing the sgRNA library was isolated via a Qiagen Plasmid Plus MaxiPrep kit (Qiagen 12963).

### Combinatorial fitness screening and NGS prep from gDNA

#### Transfection of HEK293T cells for lentivirus production

HEK293T cells were used to produce lentivirus for the pooled CRISPR screens. One day before transfection, HEK293T cells were seeded into a 15-cm dish so that they would be approximately 70–80% confluent the following day. On the day of transfection, 36 μL of Lipofectamine 2000 was added to 1.5 mL of Opti-Mem reduced serum media. In a separate 1.5 mL of Opti-Mem, 12 μg pCMVR8.74, 3 μg pMD2.G, and 9 μg of the sgRNA containing lentivector were mixed. After 5 min, the lipofectamine containing OptiMem and the diluted DNA were mixed gently and incubated at room temp for 25 min. While this was incubating, the HEK293T cells were replenished with 20 mL of fresh media. After 25 min, 3 mL of the lipofectamine/DNA was added to the cells dropwise. The cells were incubated for 48 h, after which the virus containing supernatant was collected and replaced with 20 mL fresh media. After 24 more hours, a second round of virus containing supernatant was harvested and combined with the first. Following this, a Steriflip 0.45 μm filter unit was used to remove contaminating HEK293T cells. The virus was then concentrated at 3500*g* and 4 °C using a 100K MWCO spin concentrator (Millipore UFC910096). Once the final volume was 1.5 mL or less, the virus was aliquoted and stored at −80 °C.

#### Lentiviral transduction

All cell lines used were transduced at a low MOI (< 0.4) to ensure every cell has only a single sgRNA integrated. Before doing a scaled up transduction at 1000 fold coverage, cells were transduced in a 12 well plate with varying amounts of virus to identify the appropriate amount of virus necessary. To transduce the cells, lentivirus was mixed with the necessary volume of cell culture media containing 8 μg/mL polybrene. The virus-containing media was added to the cells at 30% confluency and let incubate overnight. The following day, the virus/polybrene containing media was removed and replaced with fresh media. 48 h after transduction, the cells were changed into puromycin (2 μg/mL) containing media. Cells were then grown as normal in media containing puromycin.

#### Fitness screening in TNBC cell lines

Fitness screening was performed in three TNBC cell lines: Hs578T, MDA-MB-231, and MDA-MB-468. All cells were grown in DMEM media (Thermo 10566016) supplemented with 10% FBS (Thermo 10082147) and antibiotics/antimycotics (Thermo 15240096). Cells were passaged every 3–4 days via 0.25% Trypsin–EDTA (Thermo 25200056). The TNBC cell lines were grown for a total of 28 days, freezing down (−80 °C) aliquots of cell pellets at each passage as well as a portion of cells three days after transduction. Care was taken to ensure that the number of cells plated and frozen down were greater than 1000 fold the library size. After the completion of the screen, a Qiagen DNeasy blood and tissue kit was used to isolate genomic DNA from four evenly spaced time points over the course of the screen. After genomic DNA extraction, primers NGS_dualgRNA_SP_Lib_F and NGS_ dual-gRNA_SP_Lib_R (Supplemental File 1) were used to amplify the dual sgRNA cassette for sequencing. For each sample, 40 μg of genomic DNA was mixed with 250 μL of Kapa Hifi HotStart ReadyMix, 25 μL of each primer (10 μM stock), and water up to 500 μL. The amplification was performed according to the manufacturer’s protocol, with an annealing temperature of 55 °C and an extension time of 45 s. The step 1 PCR product was then purified using a QiaQuick PCR Purification Kit. Following this, an NEBNext indexing kit (NEB E7335S) was used to attach Illumina specific sequences and indices via nested PCR. 1 μL of the purified step 1 PCR amplicon as template (the sgRNA library) was added with 2.5 μL of each indexing primer per 50 μL Kapa HiFi reaction, and run for 6–8 cycles with an annealing temperature of 65 °C and an extension time of 45 s. The final dual sgRNA sequencing libraries were then purified using AmpureXP magnetic beads (Beckman A63881) at a 0.8:1 bead-to-DNA ratio. The libraries were subsequently sequenced with at least 500 fold sequencing coverage using a HiSeq2500 operating in rapid mode.

### Genetic interaction scoring

#### Counting gRNAs

The abundance of cells harboring dual CRISPR constructs, the fitness estimation of those constructs, and resulting interaction scores were quantified as previously described^50^ with modification. Briefly, the DNA aligner Bowtie2^100^ was used to align the sequencing reads harboring sgRNAs to a reference of expected guides and background amplicon sequence. The NGS read format of the dual CRISPR constructs was as follows:

Read1: 5′-TATATATCTTGTGGAAAGGACGAAACACCG <gRNA_1> GTTTCAGAGCTATGCTGGAAACTGCATAGCAAGTTGAAATAAGGCTAGTCC-3′.

Read2: 5′-CCTTATTTTAACTTGCTATTTCTAGCTCTAAAAC <gRNA_2> GTTTTAGAGCTAGAAATAGCAAGTTAAAATAAGG-3′.

gRNA_1 and gRNA_2 are the guide RNAs targeting gene 1 and gene 2, respectively. A reference sequence fasta sequence was constructed by prepending the 5′ sequence and appending the 3′ sequence to each guide RNA in positions 1 and 2 separately. This resulted in a reference sequence with 224 ‘contigs’ or expected sequences, 112 in each gRNA position. The bowtie2 index files were then built with the command ‘bowtie2-build’. The individual read 1 and read 2 fastq files were aligned separately with ‘bowtie2-align’ using the ‘-very-sensitive’ preset. After alignment, bam tags were added to each alignment specifying the index position of the first base of the gRNA, the expected gRNA based on which gRNA contig the read was aligned to, and the Levenshtein distance of the read to the expected guide sequence. Additionally, the bam binary flag was modified to include mate pair information. The individual read 1 and read 2 bams were then merged with ‘samtools merge’, coordinate sorted with ‘samtools sort’, and the mate pair information fixed with ‘samtools fixmate’. Guide-guide pairs were then counted from the aligned bam files. The individual reads are filtered to those with a Levenshtein distance of less than 3, allowing for a maximum of two insertions, deletions, or mismatches in the guide sequence. Furthermore, for a given mate pair to be valid, we require that each read is aligned to a contig expected in that position. The pair of guide sequences observed in read 1 and read 2 for a given mate pair are also required to be expected from the library construction. These requirements ensure we do not quantify sequencing reads or PCR errors.

The relative abundance of each dual gRNA construct, ${x}_{{g}_{1}{g}_{2}}$, was represented as a log_2_ transformed ratio of the number of reads assigned to that pair, ${M}_{{g}_{1}{g}_{2}}$, to the total number of reads assigned to any construct in the experiment:

1 $${x}_{{g}_{1}{g}_{2}}= lo{g}_{2}\frac{{M}_{{g}_{1}{g}_{2}}}{{\sum }_{i}^{N}{\sum }_{j\ne i }^{N}{M}_{{g}_{i}{g}_{j}}}$$

where $N$ is the total number of individual gRNAs. The log2 changes in abundance induced by each gRNA pair, ${m}_{{g}_{1}{g}_{2},t}$, at each timepoint $t$ was estimated as the difference between the abundance on day *t* and the abundance in the initial infection (${t}_{0}$):

2 $${m}_{{g}_{1}{g}_{2},t}= {x}_{{g}_{1}{g}_{2},t}-{x}_{{g}_{1}{g}_{2},{t}_{0}}$$

These changes in abundances ${m}_{{g}_{1}{g}_{2},t}$ were then *Z*-score standardized. The standardization serves to scale ${m}_{{g}_{1}{g}_{2},t}$ to a dimensionless number that has similar distribution across different times.

3 $${f}_{{g}_{1}{g}_{2},t}=\frac{{m}_{{g}_{1}{g}_{2},t} - {\mu }_{t}}{{\sigma }_{t}}$$

#### Scoring genetic interactions

A genetic interaction, π, was scored as the deviation in observed dual gRNA construct fitness, ${f}_{{g}_{1}{g}_{2}}$, from the multiplicative effects of the individual gRNA construct fitnesses. Since the fitness *f* is log transformed, the genetic interaction score is described as follows:

4 $${f}_{{g}_{1}{g}_{2}}= {f}_{{g}_{1}}+ {f}_{{g}_{2}}+ {\pi }_{{g}_{1}{g}_{2}}$$

The single guide effects *f*_*g1*_ (or equivalently *f*_*g2*_*, f*_*g3*_ … *f*_*gN*_) were imputed as follows. Summing Eq. (4) over all gRNA pairs containing *g*_*1*_, we have:

5 $${\sum }_{j=2}^{N} \, {f}_{{g}_{1}{g}_{i}}= (N-1){f}_{{g}_{1}}+{\sum }_{j=2}^{N}\, {f}_{{g}_{j}}+{\sum }_{j=2}^{N}{\pi }_{{g}_{1}{g}_{j}}$$

Under the assumptions that genetic interactions are rare and centered about zero^101^, the final term of this equation is dropped:

6 $${\sum }_{j=2}^{N}\, {f}_{{g}_{1}{g}_{i}}\simeq (N-1)\, {f}_{{g}_{1}}+{\sum }_{j=2}^{N}\, {f}_{{g}_{j}}$$

The set all summations for each gRNA is then solved as a system of linear equations, *Ax* = *b*, where *A* is an *N*⨉*N* matrix, *x* is the vector of single gRNA fitnesses *f*_*g*_ to be imputed, and *b* is the sum of all construct fitnesses harboring gRNA *i* (Eq. 5).

7 $$\left[ {\begin{array}{*{20}c} {N - 1} & {1 \ldots 1} & 1 \\ \vdots & \ddots & \vdots \\ 1 & {1 \ldots 1} & {N_{n} } \\ \end{array} } \right]\left[ {\begin{array}{*{20}c} {f_{g1} } \\ \vdots \\ {f_{gn} } \\ \end{array} } \right] = \left[ {\begin{array}{*{20}c} {\sum\nolimits_{j = 2}^{N} \, {f_{g1,gj} } } \\ \vdots \\ {\sum\nolimits_{j = 1}^{N - 1} \, {f_{gN,gj} } } \\ \end{array} } \right]$$

Having used this equation to impute values for each ${f}_{g}$, we then solve Eq. (4) for all genetic interaction terms ${\pi }_{{g}_{1}{g}_{2}}$.

Each pair of genes in the screening library, *a* and *b*, corresponds to 32 distinct combinations of gRNAs: each gene is targeted by 4 distinct gRNAs, resulting in 4 ⨉ 4 = 16 unique gRNA combinations per gene pair, and the gene pair appears in 2 orders (*a*,*b* or *b*,*a*). To compute gene level genetic interaction scores, we averaged π_g1,g2_ across all 32 combinations of gRNAs for a given gene pair. The gene level interaction scores were then z-score normalized for each time point in each replicate. A final estimate of the gene–gene interaction score was computed as the median z-score for all 3 timepoints and 2 replicates.

#### Validation of candidate genetic interactions

We validated candidate genetic interactions using a previously described technique^52^ as follows. sgRNA used in the screen (Supplemental File 1) were selected and cloned into the lentiviral pKLV2-U6gRNA5(BbsI)-PGKpuro vector backbone expressing either BFP or mCherry (Addgene #67974 or #67977). Cells were transduced in triplicate to create four populations, and abundance of each population was quantified by FACS Aria. Analysis was performed with Flowjo (v10.8.1).

### Single-cell RNA sequencing of pooled knockout cells

The DNA coding for each sgRNA construct was generated using two overlapping oligonucleotides containing the guide sequence and homology arms for Gibson cloning. The full list of oligonucleotides used to generate sgRNA constructs is contained in Supplemental File 1. To produce a double-stranded insert for Gibson Assembly cloning, 1 μL of each primer (10 μM) was added to 8 μL of ultrapure water and 10 μL Kapa Hifi HotStart ReadyMix. The PCR reaction was performed according to the manufacturer's protocol with an annealing temperature of 60 °C and an extension time of 15 s and 7 cycles. Following this, the sgRNA insert was purified using a QiaQuick PCR purification column. 50 ng of BsmBI digested CROP-Cas9-Puro vector was then incubated with 10 ng of purified sgRNA insert in a 10 μL Gibson Assembly reaction for 1 h at 50 °C. This Gibson reaction was then directly transformed into Stbl3 chemically competent cells according to the manufacturer's protocol. Colonies were then miniprepped and sequenced to identify correctly cloned constructs. After sequence verifying all targeting sgRNA plasmids in the library, they were quantitated via Nanodrop and pooled at equal molarity, excluding the non-targeting and AAVS1-targeting negative control guides which were included at 25% of the total library.

For scRNA-seq experiments, cells were transduced with lentivirus at 30% confluency in a 10 cm dish to maintain library coverage. After transduction (see above), cells were grown for 7 days, then processed via 10× Genomics 3′ Single Cell mRNA Capture Kit v3 according to the manufacturers protocols. Unused cDNA from the library prep was used to amplify the CRISPR sgRNA sequences to improve cell annotation. In a 50 μL reaction, 20 μL of cDNA was mixed with 2.5 μL of the CROP-Seq_Guide_Amp primer (10 μM), 2.5 μL of the NEB_Universal primer (10 μM) (Supplemental File 1), and 25 μL of Kapa HiFi HotStart ReadyMix. The PCR cycling parameters were used according to the manufacturer's protocol, with an annealing temperature of 65 °C and an extension time of 30 s. Care was taken to ensure the PCR reaction was terminated in the exponential phase by performing a small scale test PCR reaction and running several different cycle numbers on an agarose gel to visualize amplification kinetics. After amplifying and purifying the sgRNA libraries via a Qiagen PCR purification column, the libraries were then indexed for Illumina sequencing via an NEBNext multiplexed indexing oligo kit. 1 μL of the purified step 1 PCR amplicon as template (the sgRNA library) was added with 2.5 μL of each indexing primer per 50 μL Kapa HiFi reaction, and run for 6–8 cycles with an annealing temperature of 65 °C and an extension time of 45 s. The final sgRNA sequencing libraries were then purified using AmpureXP magnetic beads (Beckman A63881) at a 1.6:1 beads to DNA ratio. Resulting sequencing libraries were then sequenced on a NovaSeq according to 10× Genomics’ recommended sequencing parameters.

### Assessing sgRNA efficiency

Lentiviral transduction was used to delivery each sgRNA to Hs578T cells in separate wells of six-well plates. Transduction was performed at a high MOI, incubating the cells for 16 h in a 1:1 mix of unconcentrated viral supernatant (see lentiviral production section) and DMEM + 10% FBS (with 8 μg/mL polybrene). After 16 h of incubation, the virus containing media was replaced with fresh DMEM + 10% FBS, and after 48 h of incubation the media was replaced with DMEM + 10% FBS + 2 μg/mL puromycin. Following this, the cells were maintained in media containing puromycin for one week, at which point genomic DNA was isolated via the Qiagen DNeasy blood and tissue kit. The genomic DNA was then used as template for a set of nested PCR reactions to amplify the edited genomic region and subject it to NGS (see Supplemental File 1 for primers and details on editing rates). For each sample, 4 μg of genomic DNA was mixed with 25 μL of Kapa Hifi HotStart ReadyMix, 2.5 μL of each primer (10 μM stock), and water up to 50 μL. The amplification was performed according to the manufacturer’s protocol, with an annealing temperature of 60 °C, an extension time of 30 s, and 30–35 cycles of amplification. The step 1 PCR product was then purified using a QiaQuick PCR Purification Kit. Following this an NEBNext indexing kit (NEB E7335S) was used to attach Illumina specific sequences and indices via a nested PCR. 25 ng of the purified step 1 PCR amplicon as template was added with 2.5 μL of each indexing primer per 50 μL Kapa HiFi reaction, and run for 6–8 cycles with an annealing temperature of 65 °C and an extension time of 45 s. The final amplicons were then purified using AmpureXP magnetic beads (Beckman A63881) at a 1.6:1 bead-to-DNA ratio, and sequenced on an Illumina HiSeq2500. The online ‘CRISPResso’ tool (http://crispresso2.pinellolab.org/submission) was then used to quantify editing rates with default parameters^102^. For sgRNA “CCTCCTCCTCCGGCACCCAG”, targeting CDK13, we were unable to generate a high quality NGS compatible amplicon due to significant off-target amplification. Instead, we used the Synthego ICE analysis tool to estimate the editing rate from Sanger sequencing data. This methodology has been shown to well approximate results from NGS^103^.

### Cell-cycle phase scoring for unannotated genes

Co-expression networks were constructed using the “scanpy” and “numpy” Python packages^104^ using the Pearson correlation to quantify gene–gene similarity in expression. For each transcript of unknown cell-cycle relevance, cell-cycle phase scores were quantified by taking the mean Pearson correlation of the transcript of interest to a given set of known cell-cycle phase markers^67^. To quantify statistical significance, we identified genes which have a significantly higher mean coexpression with genes of a given phase versus all other phases, as quantified by a t-test. We then stratified transcripts by the variance in their cell-cycle phase scores, only plotting genes with cell-cycle phase scores with variance greater than 2 standard deviations away from the dataset mean.

### Cell-cycle phase annotation

#### Preprocessing read counts

The sequencing counts from the scRNA-seq experiments were quantified with CellRanger^105^, which provides estimates of mRNA abundance per gene and classification of which sgRNA each cell harbors. “Scanpy” was used for downstream processing of the mRNA expression estimates. Cells for which the mRNA samples have fewer than 200 genes expressed, or more than 10,000 genes expressed, were removed with the scanpy function “filter_cells”. Likewise, genes expressed in fewer than 3 cells were filtered from the expression matrices with the scanpy function “filter_genes”. Next, the fraction of read counts mapping to mitochondrial genes was quantified, and cells with more than 10% mitochondrial reads were removed. The expression estimates were then read-count normalized with the function “normalize_total” and log normalized with the scanpy function ‘log1p’.

#### Expression markers of cell cycle and coarse classification of cell-cycle phase

For each cell *i*, the cell-cycle phase was estimated using numpy and pandas in custom python scripts. First, we obtained five sets of genes (${J}_{k}$), $k \in K= \{M, M/G1, G1/S, S, G2/M\}$, containing genes that had been previously identified as biomarkers of discrete cell-cycle phases^69^ as well as cell-cycle biomarkers newly identified from our transcriptomic data (Supplemental File 1). For each ${J}_{k}$ we computed the average expression, ${E}_{ik}$:

8 $${E}_{ik}=\frac{{\sum }_{j\in {J}_{k}}{E}_{ij}}{|{J}_{k}|}$$

We also computed a pan-phase expression profile *E*_*i*_, with all genes implicated in any cell-cycle phase:

9 $${E}_{i}={\cup }_{\forall k}{E}_{ik}$$

These expression vectors were also used to label each cell with a coarse-grained classification *C* $\in K$ of the cell-cycle phase:

10 $${{C}_{i}=argma{x}_{k}{E}_{ik}}$$

#### Embedding of single-cell expression to quantitate cell-cycle phase angle

For each pair of cells (*m*, *n*), we computed the cosine similarity of the pan-phase expression profiles (Eq. 9), which was used to derive the pairwise cell–cell distance *D*:

11 $${D}_{m,n}=1-cos\left({\Theta }_{m,n}\right) = 1 - \frac{{E}_{m}\cdot {E}_{n}^{T}}{\left|\left|{E}_{m}\right|\right|\left|\left|{E}_{n}\right|\right|}$$

The matrix of all pairwise cell–cell distances, $D$, was then embedded into two dimensional space (${D}_{1}$ and ${D}_{2}$) using Multidimensional Scaling^106^ (MDS) in sklearn. The Cartesian coordinates of each cell in the embedding were converted to polar coordinates:

12 $$(r, \Theta )=\left(\sqrt{{{D}_{1}}^{2}+{{D}_{2}}^{2},} {tan}^{-1}\frac{{D}_{2}}{{D}_{1}}\right)$$

We then assigned consecutive angular ranges to discrete cell-cycle labels $k$ according to the ${C}_{i}$ that was most represented among the cells within that range. Defining ${S}_{\Theta }$ as the set of all cells residing in a angular range bounded by $\Theta$ and $\Theta +1$, the most represented cell-cycle phase label was:

13 $${M}_{\Theta }= argma{x}_{k}\left|{C}_{i,0}=k \forall i\in {S}_{\Theta }, k \in K\right|$$

We used linear regression to assess the ability of $\Theta$ to capture cell-cycle information and to consequently be used to remove that information from the transcriptome-wide expression profile. We first smoothed the expression estimates for each cell in each phase, ${E}_{ik},$ across the angular dimension, $\Theta$, with the R package ‘mgcv’^107^. The modified cell-cycle expression scores were then used as features in the ‘regress_out’ function in scanpy. Kuiper’s test, a Kolomogrov-Smirnov test in polar coordinates available in the R package “circular”^108^, was used to score which gene knockouts result in a significant change in distribution of cells about the cell-cycle embedding.

### Annotating phenotypic effects of CRISPR knockouts

To establish the baseline transcriptomic state, we calculated the median abundance per each transcript for all cells that received only one AAVS sgRNA. We calculated the log2 fold change in abundance from this baseline for each transcript of each cell. We then calculated the median fold change per transcript for each set of cells that had the same gene knockout. We also established a confidence interval of the median through 1000 bootstrap resampling. We embedded both the median and resampled median using multi-dimensional scaling, similar to the cell cycle phase analysis.

We also inferred the transcriptomic programs altered by the genetic perturbation. For each gene knockout, we compared the distribution of transcript abundances between the knockout cells and cells that received AAVS sgRNAs using a Mann Whitney-U test corrected for multiple hypothesis testing using the Bejamini-Hochberg procedure (FDR < 0.05). This procedure yielded a set of differentially expressed genes for each knockout. We then determined what cellular functions are perturbed by performing gene enrichment analysis against genesets from Reactome.

### Chemical validation of CDK12-PRMT5 interaction

MDA-MB-231 cells were seeded into 96-well flat bottom black wall plates in 100 μL/well of L-15 culture medium with 10% FBS and 1× Penicillin/Streptomycin added at 1500 cells per well and incubated overnight at 37C in air. PRMT5 inhibitor (PF-06939999^109^ or EPZ015666^110^) dilutions were prepared in 100% DMSO, then further diluted in complete culture media, and 11 ml was added to each well of the cell plate to reach the appropriate final concentration in 0.1% DMSO. Each dose was tested in triplicate. Plates were incubated for 3 days at 37 °C. Media and PRMT5 inhibitors were refreshed and SR4835^111^ was added to measure a dose response. SR4835 compound dilution plates were prepared in 100% DMSO starting with a 10 mM stock concentration, using a 3-pt serial dilution, then further diluted in complete culture media and added to each well of the cell plate such that the highest compound concentration tested was 10 mM final in 0.1% DMSO. Cells were incubated an additional 7 days at 37 °C, then plates were removed and assayed for viability using Cell Titer Glo reagent. Plates were read on an Envision plate reader using the luminescent filter. Viability was assessed as a percentage of DMSO control. The SynergyFinder 2.0^112^ web tool was used to calculate synergy scores for each PRMT5 inhibitor + SR4835 combination.

### 5′ transcript coverage bias

#### Exon coverage

Strand aware, base level read coverage was computed for each knockout in the MDA-MB-231 dataset from aligned bam files using the ‘genomecov’ tool in bedtools (version 2.30.0) with the ‘-bg’ and ‘-strand’ flags set. GENCODE comprehensive gene annotation for GRCh38 version 28 was used as a gene model for exon definitions. Exons categories for a given gene were defined as follows: ‘First’ exons are the 5′ most exon in any transcript, ‘Alternative First’ exons are other exons which are the 5′ most exon in any transcript but are not labeled ‘First’, ‘Last’ exons are the 3′ most exon in any transcript for a given gene, ‘Alternative Last’ exons are other exons which are the 3′ most exon in any transcript but are not labeled ‘Last’, ‘Internal’ exons are all other exons. Coverage per exon per gene was computed for all genes using the GENCODE annotation, based on the number of reads that span the exon with at least one base-pair, using the package bx-python (version 0.8.11). Genes with less than 10 assigned reads were filtered out. Exon coverages were subsequently normalized as reads per million and log_2_ transformed. Log_2_ fold-change per exon per gene was computed relative to cells harboring non-targeting control (NTC) guides. Significant perturbation to the fold enrichment of ‘First’ exons across the distribution of all genes measured in the scRNAseq experiment was computed using a t-test with the python package scipy (version 1.6.2).

#### Gene set enrichment of 5′ biased transcripts

The 5′ coverage bias was defined as the ratio of the fold enrichment relative to NTC of the ‘First’ exon to the ‘Last’ exon. We performed hierarchical clustering of the Euclidean distances of the 5′ bias for select knockout samples across all genes with ten or more read counts measured in the scRNAseq experiment using the ‘complete’ option from the ‘hierarchy’ package in scipy. The hierarchy was then cut into 12 trees and gene set enrichment was performed on the transcripts within each tree using the Enrichr^113^ webtool. Significantly enriched terms from the MSigDB Hallmark 2020 gene sets were determined based on p_adj_ < 0.05 by Benjamini–Hochberg corrected Fisher exact test.

### RNA Pol II transcriptional profiling via CUT&Tag

To quantify RNA pol II transcriptional initiation/activity across the genome, we employed a CUT&Tag (ActiveMotif #53165 and #91152) assay^82^. To target RNAPII, we used an antibody raised against a synthetic “YSPTSpPS” peptide corresponding to the Ser-5 phosphorylated RNAPII C-terminal domain (ActiveMotif # 91152). We used a clonal doxycycline inducible dCas9-KRAB MDA-MB-231 cell line to control repression of CDK genes and PRMT5. On day 1 of the experiment, cells were infected with lentiviruses containing the appropriate targeting/NTC sgRNAs (see Supplemental File 1), driven by the human U6 promoter at an MOI of ~ 3 for each virus to ensure all cells were transduced. Cells were transduced in DMEM + 10% FBS with the addition of 8 μg/mL polybrene. 16 h after the time of transduction, media was changed to DMEM + 10% FBS. 24 h after this, the cell culture media was switched to DMEM + 10% FBS containing 2 μg/mL puromycin to ensure no uninfected cells remain. 48 h after this, cell culture media was changed to DMEM + 10% FBS containing 2 μg/mL puromycin and 1 μg/mL doxycycline to induce dCas9-KRAB expression. 48 h after this, cells were processed for CUT&Tag library prep following the manufacturer's recommendations. To summarize, for each sample 500 K cells were spun down at 500*g* for 3 min in a 1.5 mL Eppendorf tube. The cell pellet was then resuspended in 1 mL 1× wash buffer. The cells were again spun down at 500*g* for 3 min, and resuspended in 1.5 mL 1× wash buffer. Concanavalin A beads were prepared by mixing 20 μL of beads with 1.6 mL 1× binding buffer. The tube was placed on a magnetic separator, until the beads were adhered to the wall of the tube. The supernatant was aspirated, and the beads were washed with 1.5 mL 1× binding buffer. After this, the supernatant was again removed and the tubes were removed from the magnetic rack and beads resuspended in 20 μL of 1× binding buffer. The resuspended beads were then added to cells, and allowed to mix end-over-end for 10 min at room temp. The samples were then placed on a magnetic rack, and after the beads had adhered to the wall of the tube the supernatant was removed. The cells/beads were then resuspended in 50 μL of ice-cold antibody buffer (containing protease inhibitors and digitonin), and 1uL of anti-RNAPII primary antibody was added to the samples. The primary antibody was allowed to bind overnight at 4 °C on an orbital rotator. The next day, the tubes were placed back on the magnetic rack, and the supernatant was removed after the beads had adhered to the wall of the tube. 100 µL of rabbit anti-mouse secondary antibody (diluted 1:100 in Dig-Wash buffer) was added to each tube, and allowed to bind for 1 h on an orbital rotator at room temp. Using the magnetic separator, the bead/cells were then washed three times with 1 mL of Dig-Wash buffer. The assembled pA-Tn5 transposomes were then mixed with Dig-300 Buffer at a final concentration of 1:100 (100 µL total volume). For each sample, the cells/beads were resuspended in 100 µL of the assembled transposome buffer and incubated at room temperature for 1 h on an orbital rotator. After this, the cells/beads were then washed three times with 1 mL of Dig-300 buffer via the magnetic separator. After the final wash, the supernatant was removed and the samples resuspended in 125 µL of tagmentation buffer. The samples were then incubated for one hour at 37 °C. Following this, we added 4.2 µL of 0.5 M EDTA, 1.25 µL of 10% SDS, and 1.1 µL of Proteinase K (10 mg/mL) to each sample. After mixing well, the samples were incubated at 55 °C for 1 h. The beads/samples were then placed on a magnetic separator, and the supernatant was moved to a new tube for DNA purification. 625 µL of DNA purification binding buffer was then added to each sample. The samples were then placed in a DNA purification column and spun down at 17,000*g* for 1 min. Following this, the column was washed once with 750 µL of DNA wash buffer. The column was allowed to air dry for 1 min, and then the DNA was eluted with 35 µL of elution buffer. 30 µL of the eluted DNA was then used as template for a PCR, attaching Illumina specific adapters and indices via Q5 polymerase. The PCR conditions were: 72 °C for 5 min, 98 °C for 30 s, 14 cycles of: {98 °C for 10 s, 63 °C for 10 s} followed by a final incubation at 72 °C for 1 min and a hold at 10 °C. The PCR reaction was then cleaned up using SPRI beads at a 1.1:1 beads to sample volume ratio, washing the beads twice with 200 µL of 80% ethanol. The DNA was finally eluted in 20 µL of DNA purification buffer, and the libraries sequenced on a NovaSeq 6000.

### Quantifying RNA Pol II transcriptional activity from CUT&Tag data

Adapter sequences were trimmed from the raw FASTQ files with Trim Galore using default settings and cutadapt (version 4.1). Trimmed FASTQ files were aligned with bowtie2 (version 2.4.5) with the following settings: ‘-end-to-end -very-sensitive -no-mixed -no-discordant -I 70 -X 700’. Aligned bam files were coordinate sorted and duplicates were removed with Picard Tools (version 2.17.11). Alignments with a quality score less than 2 were removed with samtools (version 1.15.1). Genomic read coverage was computed with the ‘bamCoverage’ utility in deeptools (version 3.5.1) with a binsize of 1 base pair. Read coverage across the transcript body was computed with the ‘computeMatrix’ utility in deeptools in ‘reference-point’ mode with the following settings ‘-referencePoint TSS -beforeRegionStartLength 2000 -binSize 10 -metagene -afterRegionStartLength 2000’. Read coverage values in bins across the transcript bodies were summed across all transcripts with a minimum read count of 100 and a maximum read count of 10,000. The transcriptome wide gene body coverages were normalized relative to the mean of double non-targeting control (NTC-NTC) knockouts. Significance was quantified with a Kolmogorov–Smirnov test of the mean of replicate knockdowns in the python package scipy (version 1.6.2).

## Supplementary Information

Supplementary Tables.

Supplementary Figures.

## Data Availability

All datasets and materials generated in this study are available from the corresponding author on reasonable request. Raw sequencing data has been made publicly available via the NCBI Gene Expression Omnibus and Short Read Archive (accessions: GSE218629 GSE227432, PRJNA945412). The genetic interaction scoring is available on GitHub (https://github.com/bpmunson/ctg). All other custom code, including the single-cell cell-cycle analysis, will be made available on a second a public GitHub repository (Multimodal-perturbation-analyses-of-cyclin-dependent-kinases/). The double knockout counts for each cell line in the interaction screen are included in Supplemental File 1. The network shown in Fig. 2d is stored on NDEx an open-source project for network data exchange (UUID: 30404eda-14f4-11ed-ac45-0ac135e8bacf).
