# Supplementary material for: Multimodal perturbation analyses of cyclin-dependent kinases reveal a network of synthetic lethalities associated with cell-cycle regulation and transcriptional regulation
Source: Sci Rep. 2023 May 11;13:7678. doi: 10.1038/s41598-023-33329-2 (PMC10175263; doi:10.1038/s41598-023-33329-2)
Supplement: Supplementary file 2 — Supplementary Figures. [file 41598_2023_33329_MOESM2_ESM.pdf]

SUPPLEMENTAL MATERIAL

Multimodal perturbation analyses of cyclin-dependent kinases reveal a network of synthetic lethalities associated with cell-cycle regulation and transcriptional regulation

Kyle Ford[1,*], Brenton Munson[1,2,*], Samson Fong[1,2,*], Rebecca Panwala[1], Wai Keung Chu[1], Joseph Rainaldi[1,3], Nongluk Plongthongkum[1], Vinayagam Arunachalam[4], Jarek Kostrowicki[4], Dario Meluzzi[2], Jason F. Kreisberg[2], Kristen Jensen-Pergakes[4], Todd VanArsdale[4], Thomas Paul[4], Pablo Tamayo[2], Kun Zhang[1], Jadwiga Bienkowska[4,†], Prashant Mali[1,†], Trey Ideker[1,2,†]

[1] Department of Bioengineering, University of California San Diego, La Jolla CA 92093, USA.

[2] Department of Medicine, University of California San Diego, La Jolla CA 92093, USA.

[3] Biomedical Sciences Program, University of California San Diego, La Jolla CA 92093, USA.

[4] Pfizer Inc, 10555 Science Center Drive, San Diego, CA 92121, USA.

* These authors contributed equally to this work

† Correspondence: Jadwiga.R.Bienkowska@pfizer.com, pmali@ucsd.edu, tideker@health.ucsd.edu

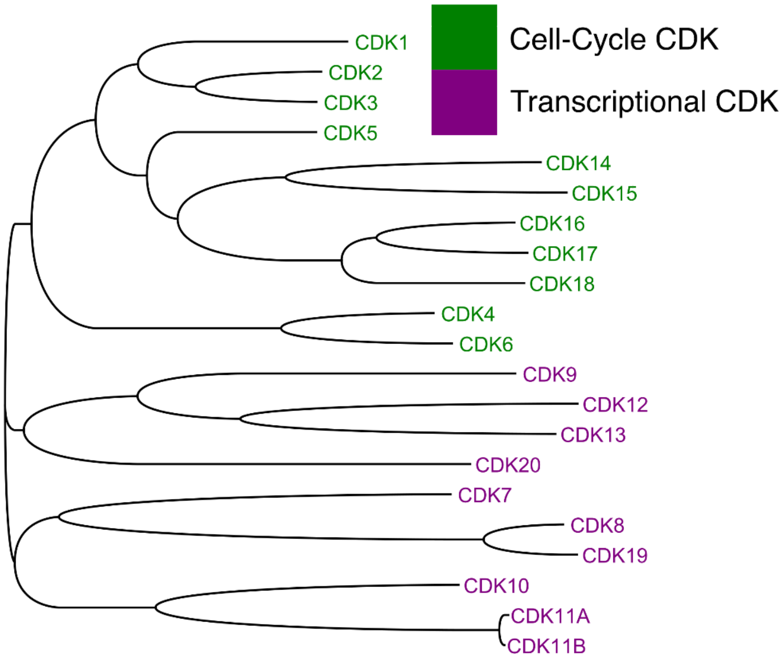

Extended Figure 1. Classes of CDK genes. Phylogenetic tree showing evolutionary relationships among CDK proteins. Tree derived from multi-sequence alignment of CDK protein amino-acid sequences (**Methods**).

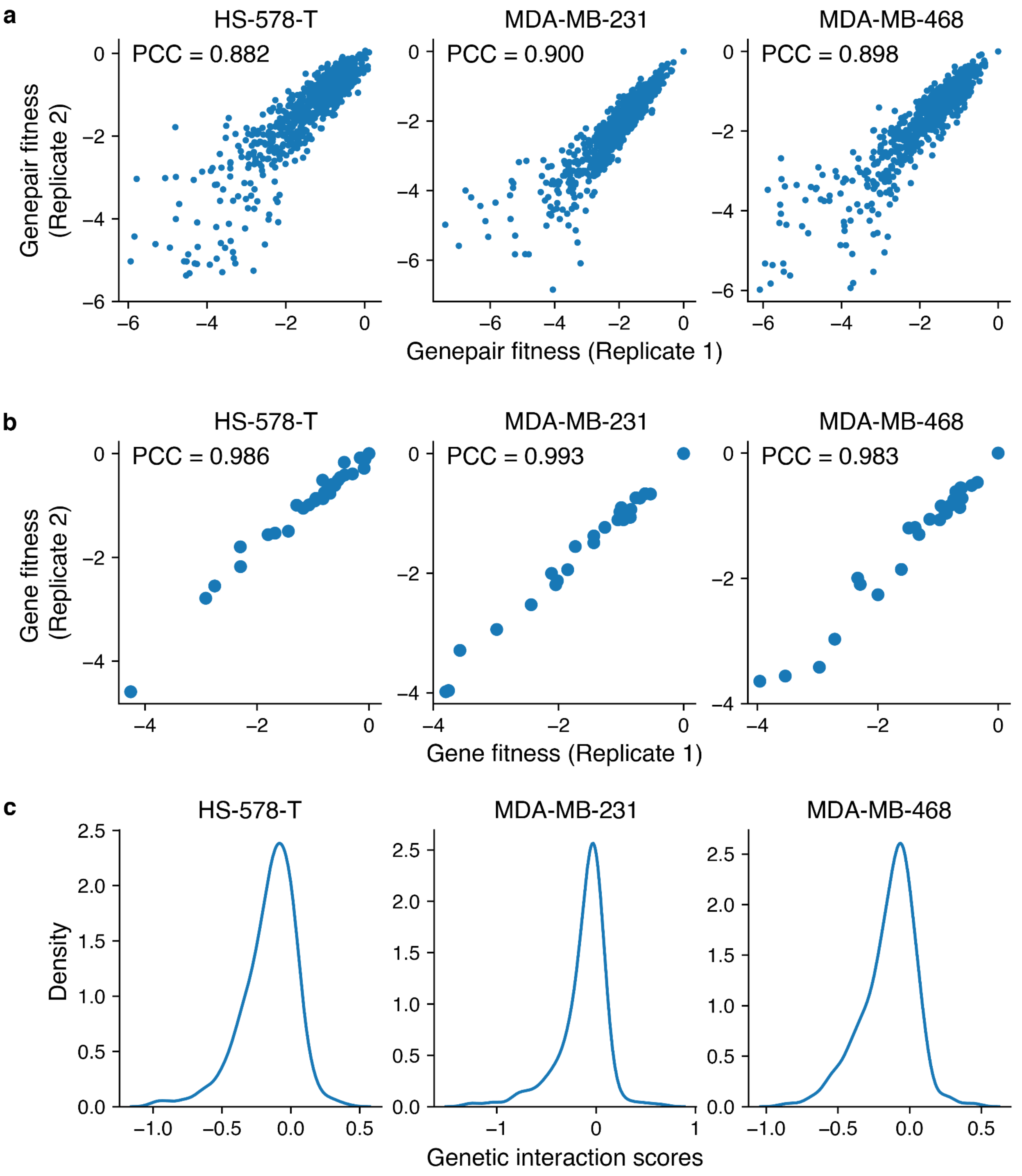

Extended Figure 2. (**a**) Genepair fitness and (**b**) gene fitness measurements across the two replicates for HS-578-T, MDA-MB-231, and MDA-MB-468. (**c**) Density distribution of genetic interaction scores for HS-578-T, MDA-MB-231, and MDA-MB-468. The mean for the three sets of genetic interaction scores are near zero.

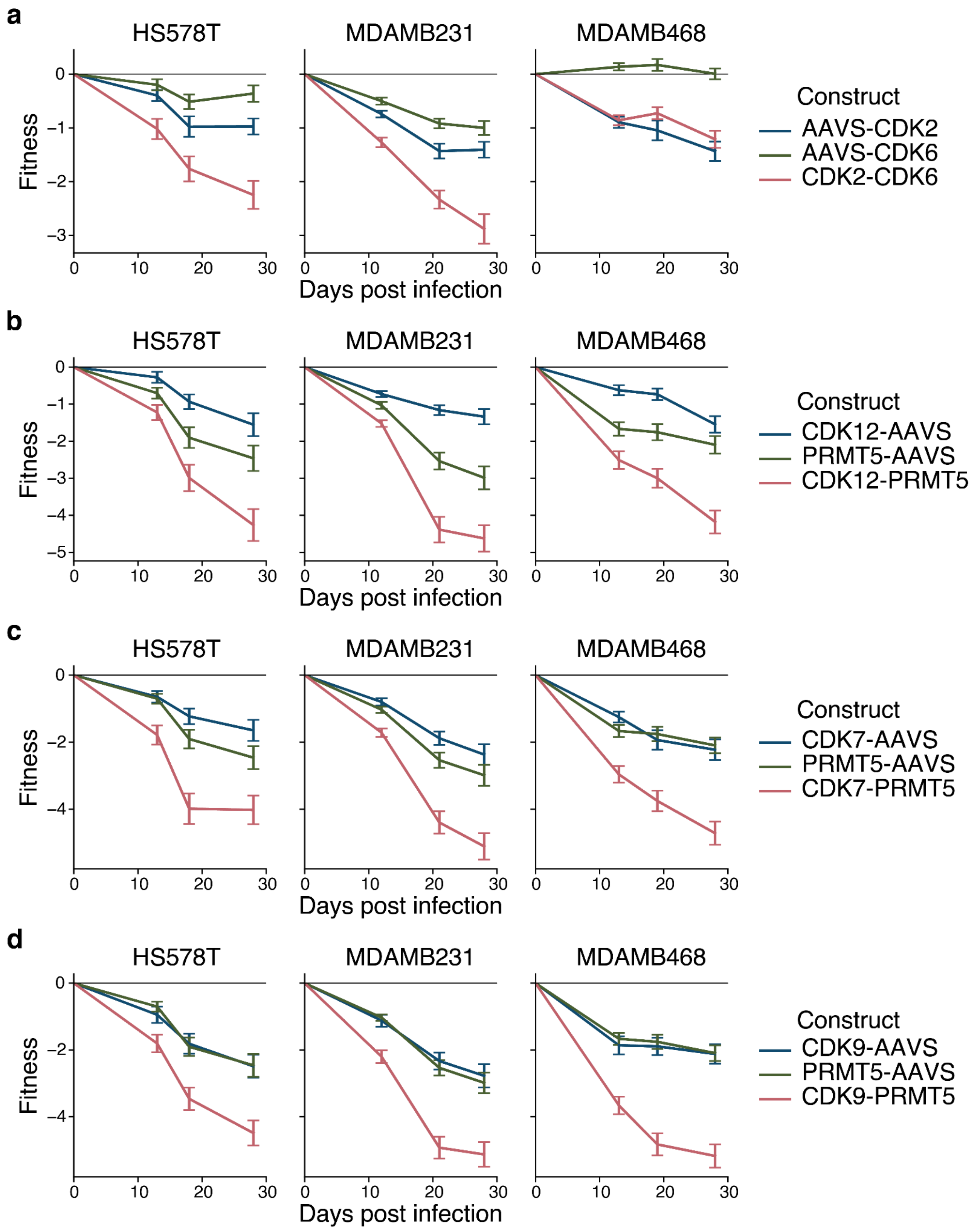

Extended Figure 3. Synthetic lethality of select double knockouts. Fitness trajectories of synthetic-lethal interactions for (**a**) CDK2-CDK6, (**b**) CDK12-PRMT5, (**c**) CDK7-PRMT5, and (**d**) CDK9-PRMT5, comparing dual knockout vs. single knockouts in HS578T, MDAMB231, and MDAMB468 cell lines (colors). Error bars correspond to fitness measurements across replicates and guide pairs targeting the same gene pair.

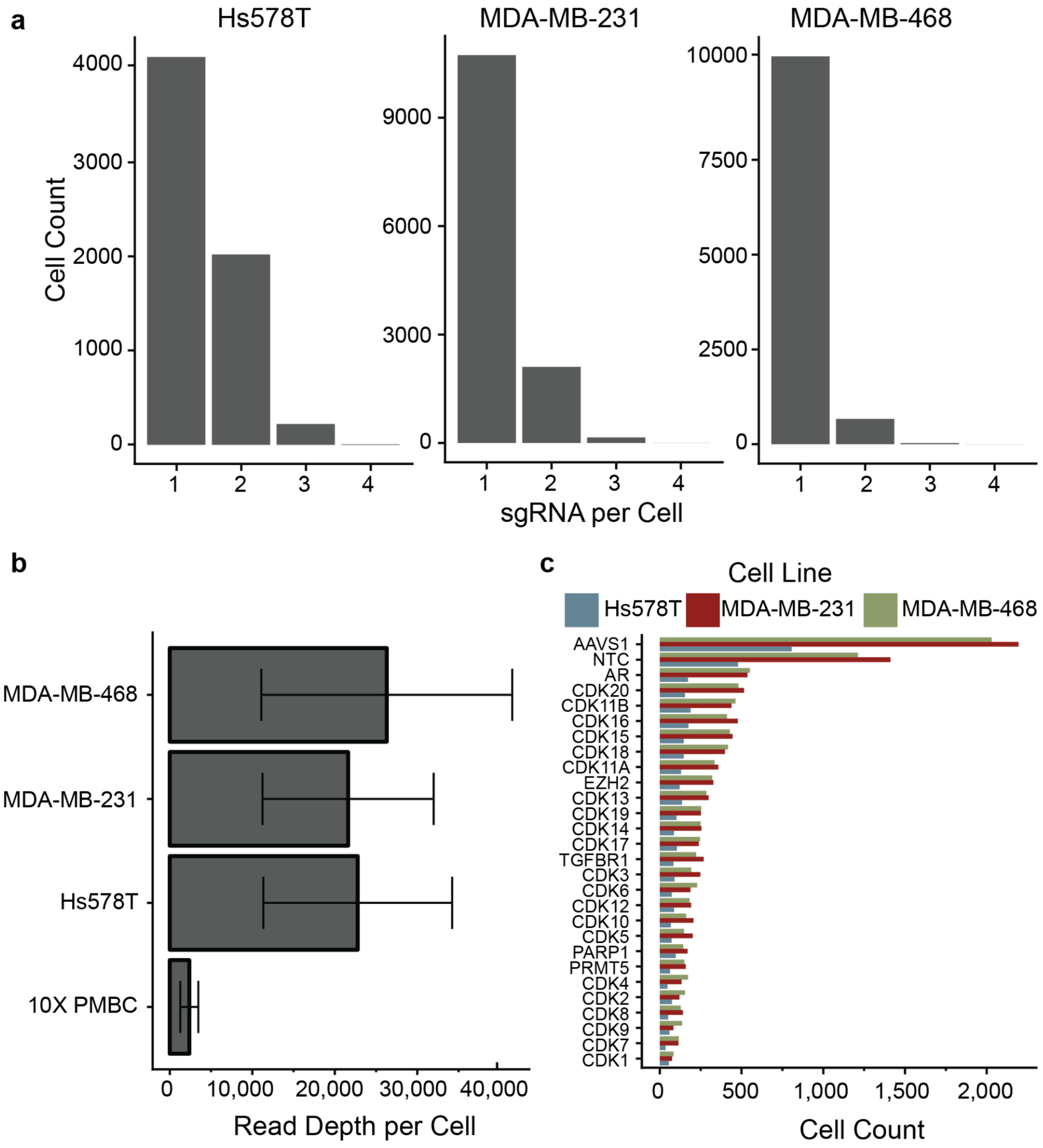

Extended Figure 4. ScRNA-seq quality control metrics. a, Histogram of sgRNA counts per cell, for each of the three cell types interrogated in this study. **b**, Read depth per cell in each cell line (10X PMBC). **c**, Histogram of number of cells receiving specific sgRNAs. AAVS1, sgRNA targeting the adeno-associated virus integration site 1, a safe-harbor locus; NTC, non-targeting control.

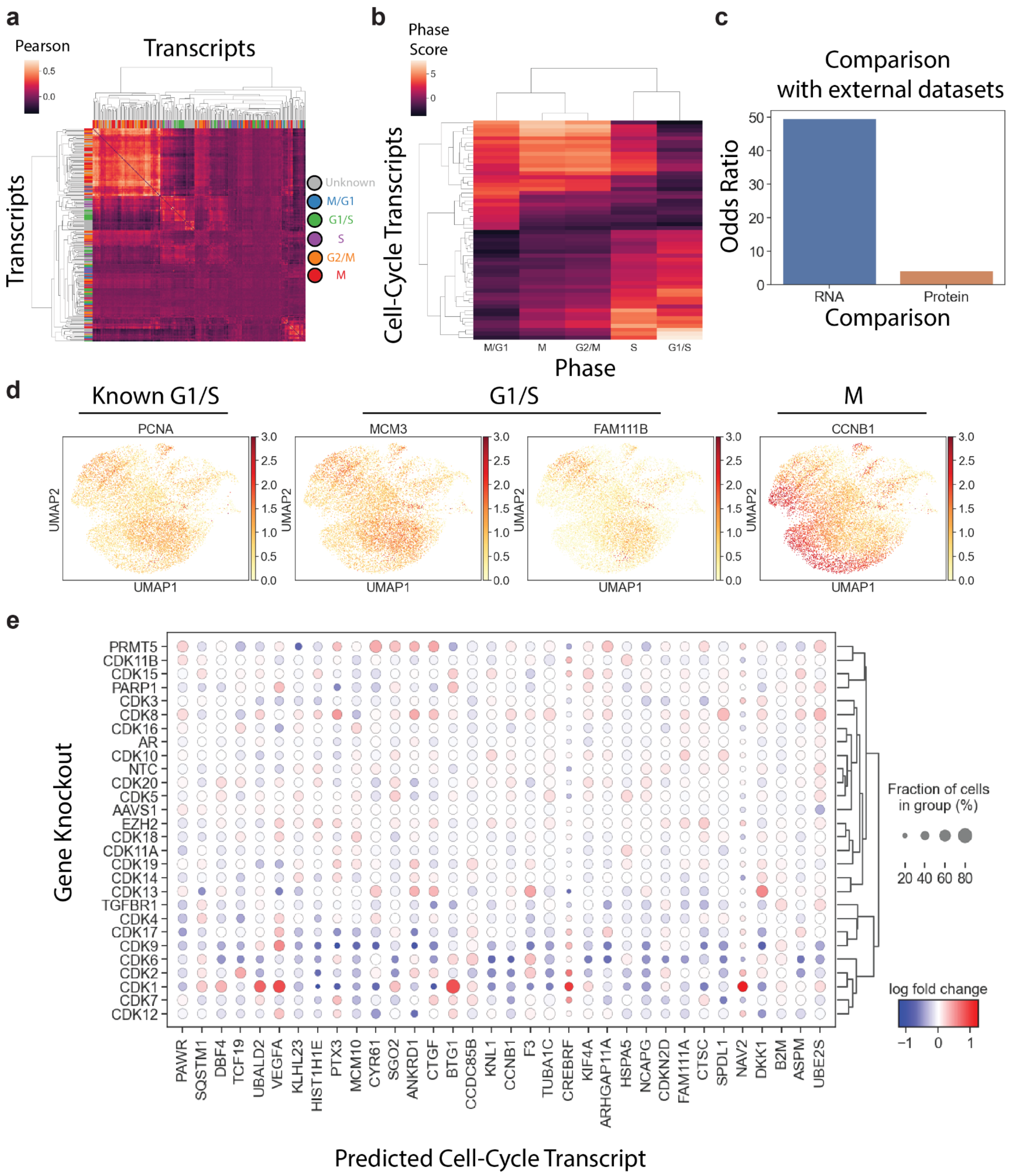

Extended Figure 5. Coexpression analysis to identify cell-cycle associated genes. a, Heatmap showing the Pearson correlation in expression for pairs of genes. MDA-MB-231 cells, highly variable transcripts only. Known cell-cycle markers marked in color on the heatmap border. **b**, Cell-cycle phase scores for predicted cell-cycle genes, defined as genes without previous phase assignment but that have significantly high correlation with marker genes of a particular phase (versus markers from all other phases, $p<0.05$). **c**, Comparison of newly identified cell cycle genes to existing datasets describing cell-cycle variable RNAs and

proteins[68]. **d**, UMAP plots showing expression levels of two predicted G1/S phase markers (MCM3, FAM111B) alongside the known marker PCNA. M-phase marker CCNB1 shown for comparison. **e**, Expression levels for identified cell-cycle genes (columns) grouped by CDK knockout (rows). Genes with significant (FDR adjusted $p<0.05$) dysregulation in response to one or more CDK knockouts are shown. Color indicates $\log_2$ fold change for each transcript relative to the population mean.

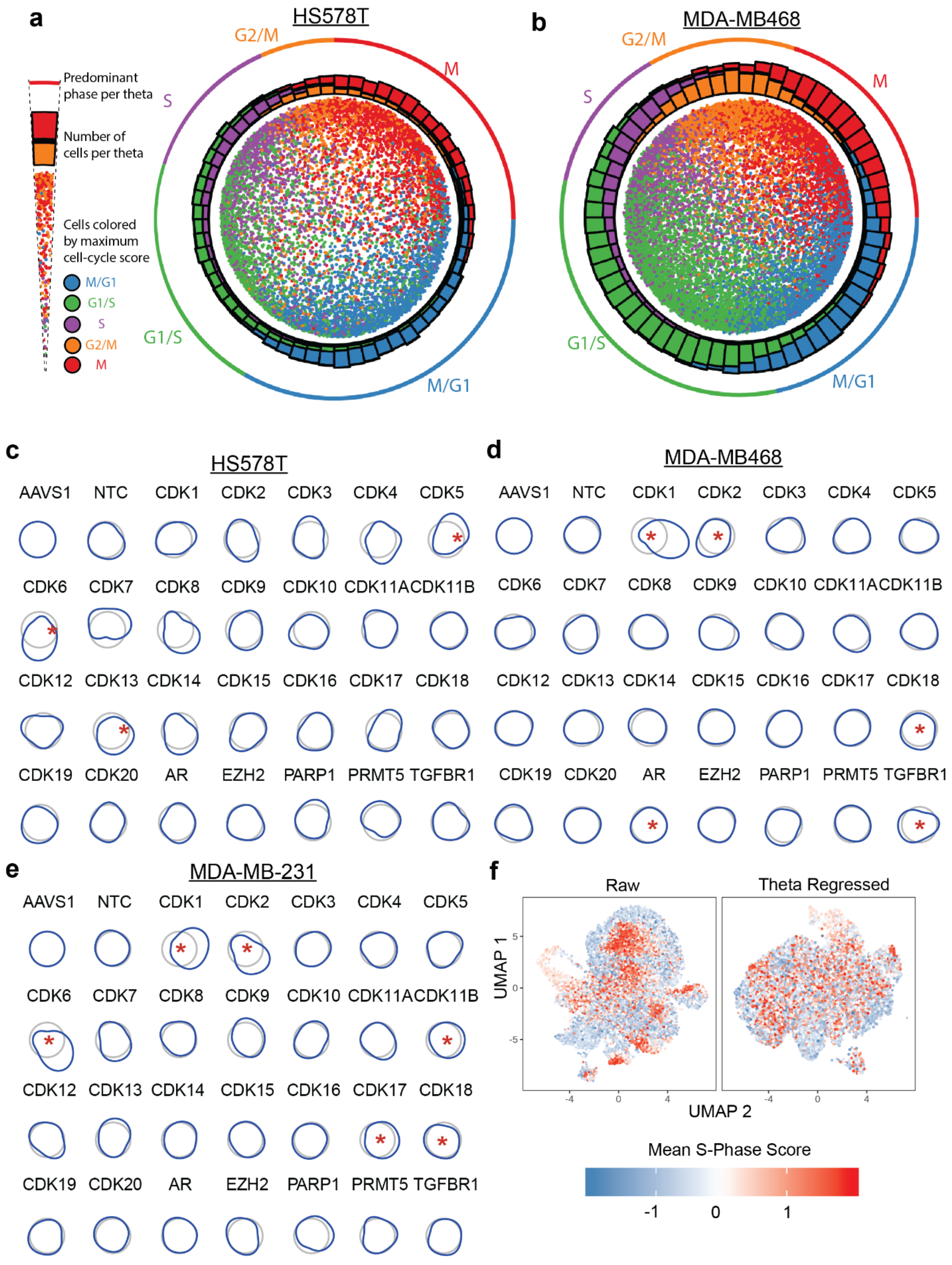
a
HS578T
G2/M
M
S
G1/S
M/G1
Predominant phase per theta
Number of cells per theta
Cells colored by maximum cell-cycle score
M/G1
G1/S
S
G2/M
M
b
MDA-MB468
c
HS578T
AAVS1 NTC CDK1 CDK2 CDK3 CDK4 CDK5
CDK6 CDK7 CDK8 CDK9 CDK10 CDK11A CDK11B
CDK12 CDK13 CDK14 CDK15 CDK16 CDK17 CDK18
CDK19 CDK20 AR EZH2 PARP1 PRMT5 TGFBR1
d
MDA-MB468
e
MDA-MB-231
f
Raw
Theta Regressed
UMAP 1
UMAP 2
Mean S-Phase Score
-1
0
1

Extended Figure 6. Cell-cycle embedding, perturbation, and regression. a, MDS cell-cycle embedding of all Hs578T cells. **b**, MDS cell-cycle embedding of all MDA-MB-468 cells. **c-e,** Deviation in single-cell density compared to AAVS for select knockouts in Hs578T (c), MDA-MB-468 (d), and MDA-MB-231 (e) cells; * $p<0.05$ by Kuiper's Test. **f**, UMAP projection of single cells before and after regression of cell-cycle phase (theta) from expression estimates; color corresponds to mean expression scores in S-phase genes after preprocessing.

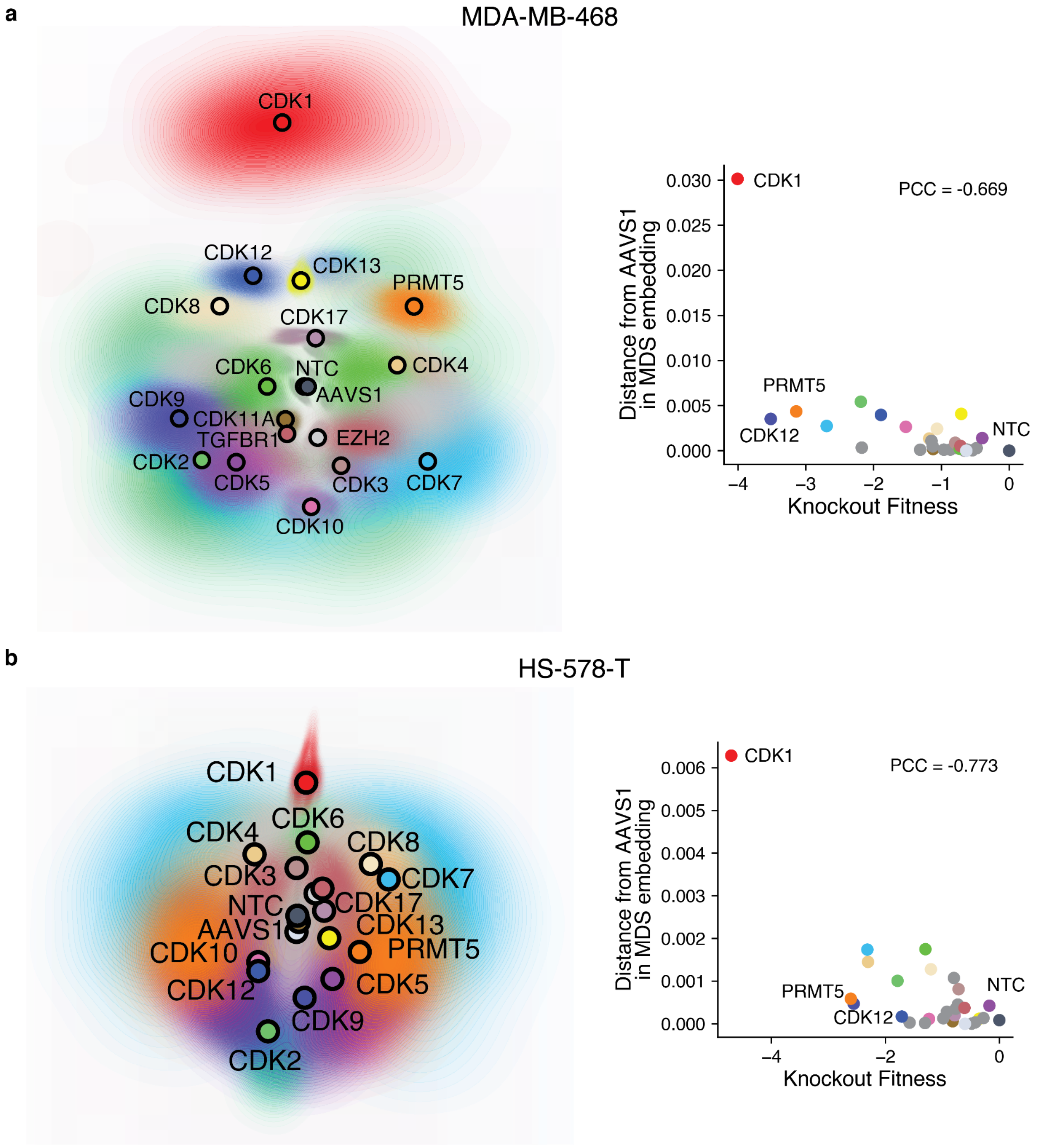

Extended Figure 7. Effects of CDK disruption on diverse transcriptional programs. MDS embedding of median single cell profile for each gene knockout for MDA-MB-468 (**A**) and HS-578-T (**B**). Each contour line depicts the confidence interval across 1,000 bootstrap resamplings. The outermost contour line represents the 95% confidence interval. For each gene knockout (colored points), the distance of the transcriptome from the AAVS control (y-axis) is plotted versus its fitness.

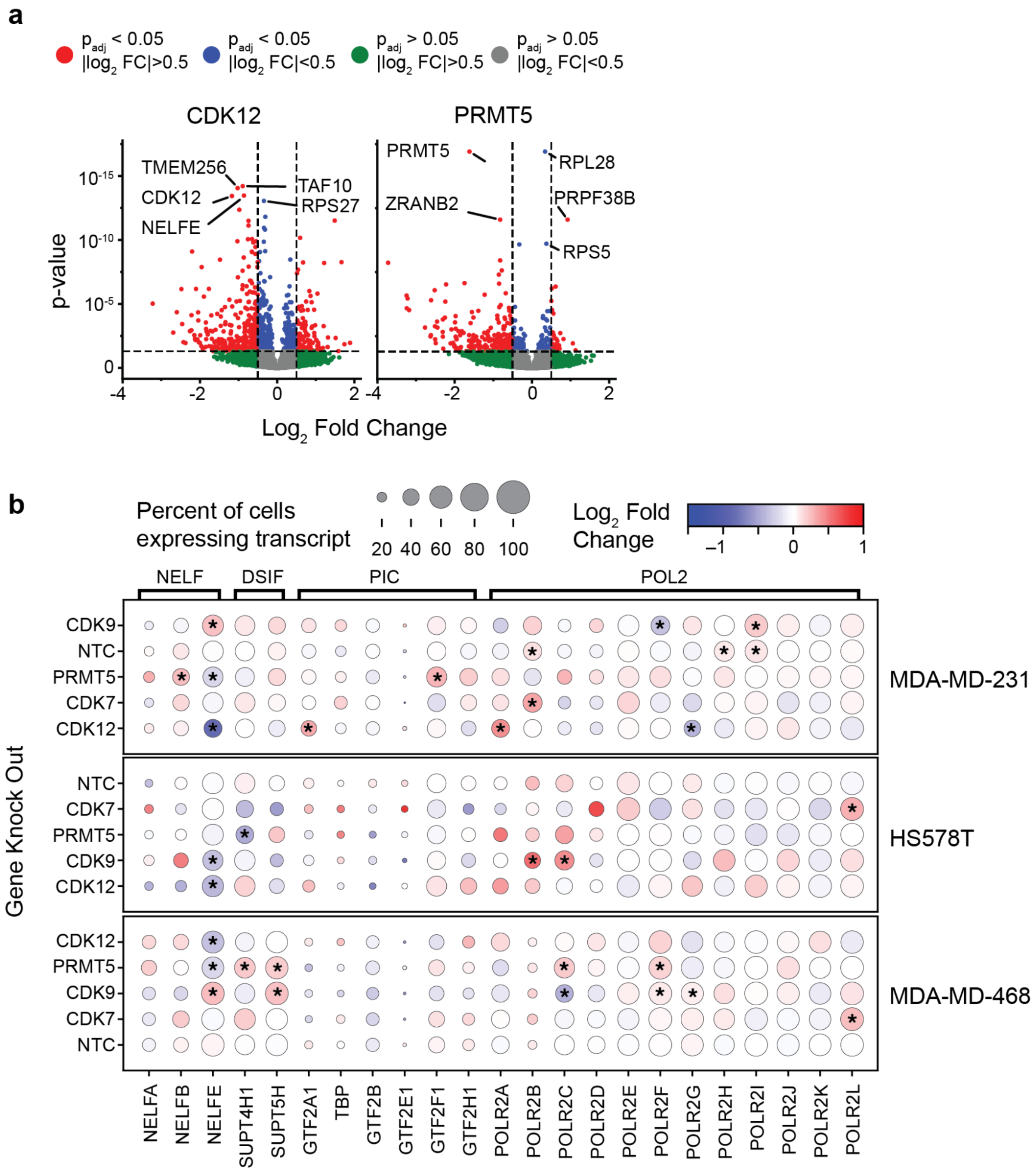

Extended Figure 8. Analyses of PRMT5 and RNAPII-associated CDKs. a, Volcano plots showing the significance vs. change in mRNA abundance level for detectable transcripts under CDK12 (left) or PRMT5 (right) knockout. The five most significantly downregulated genes are NELF, DSIF, PIC, and RNA Pol II complex members (columns)(, for select knockouts in MDA-MB-231, HS578T, and MDA-MB-468 (rows); * p<0.05 Mann Whitney-U test.

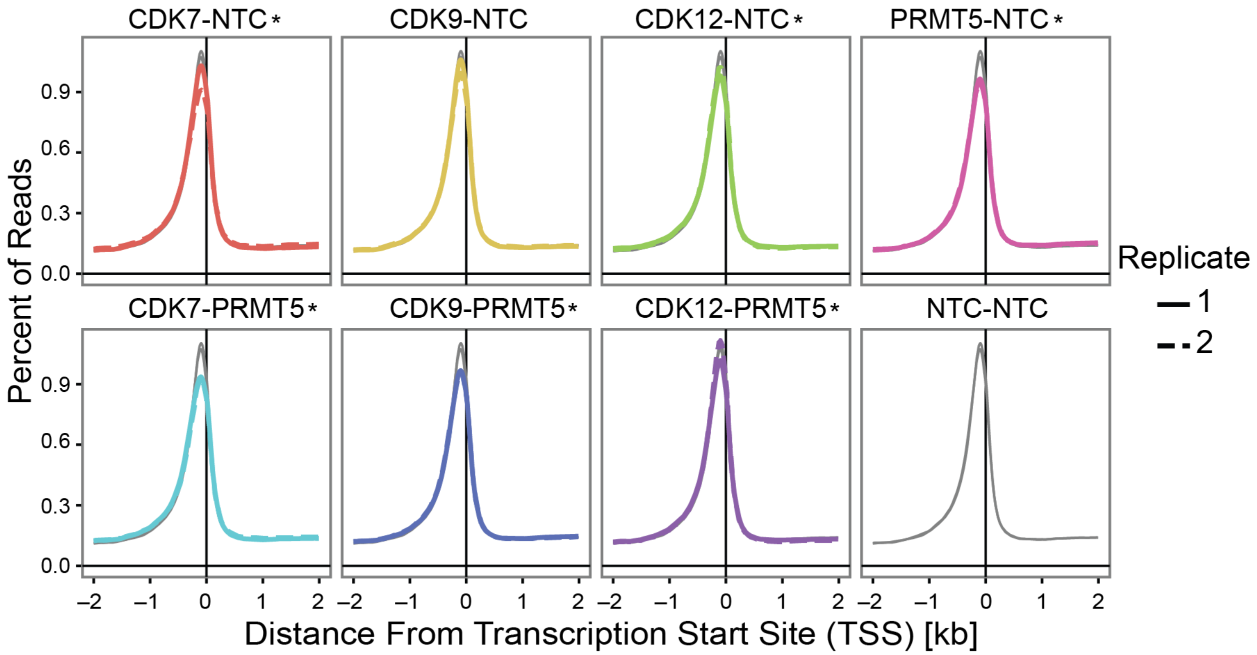

Extended Figure 9. RNA-polymerase II activity in CDK knockouts. Coverage of the location of RNA-polymerase II in the transcript body averaged across all genes measured with CUT&Tag experiments. Each of CDK7, CDK9, CDK12, and PRMT was disrupted in combination with a non-targeting control (NTC) (top row). CDK7, CDK9, and CDK12 were also disrupted in combination with PRMT5 (bottom row); combinatorial disruption using two NTCs is also shown (bottom right), as well as, in each panel in grey. Coverage profiles means for each set of replicates are compared to the mean of NTC-NTC replicates; * $p<0.05$ Kolmogorov–Smirnov test.
